# Vaccine hesitancy: evidence from an adverse events following immunization database, and the role of cognitive biases

**DOI:** 10.1186/s12889-021-11745-1

**Published:** 2021-09-16

**Authors:** Hossein Azarpanah, Mohsen Farhadloo, Rustam Vahidov, Louise Pilote

**Affiliations:** 1 John Molson School of Business, Concordia University, 1450 Guy St, Montreal, Quebec H3H 0A1 Canada; 2 Centre for Outcomes Research and Evaluation, McGill University, 5252 De Maisonneuve Blvd, Montreal, Quebec H4A 3S5 Canada

**Keywords:** Vaccine hesitancy, Adverse event, Adverse events following immunization (AEFI), Cognitive bias, Vaccine adverse event reporting system (VAERS)

## Abstract

**Background:**

Vaccine hesitancy has been a growing challenge for public health in recent decades. Among factors contributing to vaccine hesitancy, concerns regarding vaccine safety and Adverse Events (AEs) play the leading role. Moreover, cognitive biases are critical in connecting such concerns to vaccine hesitancy behaviors, but their role has not been comprehensively studied. In this study, our first objective is to address concerns regarding vaccine AEs to increase vaccine acceptance. Our second objective is to identify the potential cognitive biases connecting vaccine hesitancy concerns to vaccine-hesitant behaviors and identify the mechanism they get triggered in the vaccine decision-making process.

**Methods:**

First, to mitigate concerns regarding AEs, we quantitatively analyzed the U.S. Vaccine Adverse Event Reporting System (VAERS) from 2011 to 2018 and provided evidence regarding the non-severity of the AEs that can be used as a communicable summary to increase vaccine acceptance. Second, we focused on the vaccination decision-making process. We reviewed cognitive biases and vaccine hesitancy literature to identify the most potential cognitive biases that affect vaccine hesitancy and categorized them adopting the Precaution Adoption Process Model (PAPM).

**Results:**

Our results show that the top frequent AEs are expected mild reactions like injection site erythema (4.29%), pyrexia (3.66%), and injection site swelling (3.21%). 94.5% of the reports are not serious and the average population-based serious reporting rate over the 8 years was 25.3 reports per 1 million population. We also identified 15 potential cognitive biases that might affect people’s vaccination decision-making and nudge them toward vaccine hesitancy. We categorized these biases based on the factors that trigger them and discussed how they contribute to vaccine hesitancy.

**Conclusions:**

This paper provided an evidence-based communicable summary of VAERS. As the most trusted sources of vaccine information, health practitioners can use this summary to provide evidence-based vaccine information to vaccine decision-makers (patients/parents) and mitigate concerns over vaccine safety and AEs. In addition, we identified 15 potential cognitive biases that might affect the vaccination decision-making process and nudge people toward vaccine hesitancy. Any plan, intervention, and message to increase vaccination uptake should be modified to decrease the effect of these potential cognitive biases.

**Supplementary Information:**

The online version contains supplementary material available at 10.1186/s12889-021-11745-1.

## Background

Vaccine hesitancy that is defined by the Strategic Advisory Group of Experts (SAGE) on immunization as “delay in acceptance or refusal of vaccines despite availability of vaccination services” [1] has always been a public health threat. It has always had significant consequences, such as the resurgence of once eradicated vaccine-preventable diseases or failure to achieve or sustain herd immunity [1]. The current COVID-19 pandemic and the public reaction to the COVID-19 vaccine is another example of this substantial challenge. Although the scientific community puts an unprecedented global effort to end the COVID-19 pandemic, vaccine hesitancy might interfere with these efforts. In a survey on U.S. adults, 10.8% indicated they would refuse COVID-19 vaccination, and 33.6% stated they were unsure about accepting COVID-19 vaccinations [2]. Other survey studies also show that 20% of people in the U.S. and 26% of French adults will decline the COVID-19 vaccine [3, 4], which may cause failure to generate herd immunity [3].

Vaccine hesitancy is a complex and context-specific challenge varying across time, place, and vaccines. Factors contributing to vaccine hesitancy lie over a broad spectrum, from the risk-benefit of vaccination concerning vaccine safety and Adverse Events (AE) and knowledge and awareness issues to religious, cultural, gender, socio-economic, and vaccine-specific factors [5–8]. In an attempt to validate the vaccine hesitancy scale developed by the SAGE working group on vaccine hesitancy, lack of confidence and risks have been found as two factors constructing such scale [9]. While the lack of confidence incorporates the importance and effectiveness of vaccines and reliability and trustworthiness of the vaccine information sources, perceived risk is about the lack of trust in vaccine safety and concerns regarding Adverse Events Following Immunization (AEFI).

Lack of trust in vaccine safety and concern regarding AEFIs plays a significant role in vaccine-hesitancy [5–7, 10–13] in two levels: general vaccine hesitancy or a specific vaccine hesitancy, which both are associated with lower vaccine uptake [10]. Two systematic reviews investigating factors affecting general vaccine uptake [11] and the seasonal-influenza-vaccine uptake [12] show a strong association between vaccine uptake and perception of vaccine safety and AEFIs. That is the same concern for the COVID-19 vaccine as people distrust its safety at the COVID-19 vaccine level and the general level [2, 3]. However, most of the respondents who were unsure about accepting the COVID-19 vaccine (31.6%) in [2] indicated that they might accept vaccination if they receive credible information about its safety and effectiveness. This points to the lack of communication about vaccine information, including their AEs and building trust in immunization programs that is another factor associated with vaccine uptake [5, 7, 11, 12].

Distrust in vaccine safety and AEs and the lack of effective communication about vaccine information demand reconstructing vaccine communication. Harrison and Wu [13] suggest that increasing public trust in vaccines needs reshaping medicine’s cultural environment to communicate better the risks and benefits of vaccines. One way to achieve this is by providing meaningful, evidence-based information on the known risks of vaccination and acknowledging the unknown risks [14]. Vaccine AE databases can be used as a source for evidence-based communication about vaccine safety and AEs. Databases such as the U.S. Vaccine Adverse Events Reporting System (VAERS) that receive voluntary submissions of suspected AEFIs by individuals who experience them have been established for postmarketing monitoring of vaccination AEs, detecting new, unusual, or rare vaccine AEs, and assessing the risk of newly licensed vaccines [15]. Using VAERS data, Scherer, Shaffer [16] have tested the effect of different forms of open communications on people’s acceptance and trust in HPV vaccination and have found that augmenting vaccine information with a summary report from VAERS significantly increases trust and acceptance of vaccines.

A critical point in vaccine communication and decision-making is the role of cognitive biases in this process. When making a decision, people often use heuristics that simplify the problem domain. These heuristics are useful if they get triggered by the right factors. However, when people use these heuristics under the influence of wrong factors, they lead to systematic errors, i.e., cognitive biases [17, 18]. For example, the availability heuristic, which is the tendency to attribute more weights to factors that are easier to recall, is a valid ecological clue for the judgment of frequency as more frequent events are easier to recall [18]. However, the availability of an emotionally compelling story about a rare AE might cause parents to perceive that rare incident as a frequent AE and nudges them towards vaccine hesitancy. In that case, it is a cognitive bias. When Blaisdell, Gutheil [14] suggest providing evidence-based information on the risks of vaccination and acknowledging the unknown risks to increase trust in vaccination, they actually refer to reconstruing two factors associated with vaccination: perceived risks and perceived ambiguity. These two factors can trigger several cognitive biases, including omission bias [19], loss aversion [20], ambiguity aversion [21], optimism bias [22], and present bias [22] that might nudge vaccine-decision makers (parents/patients) toward vaccine refusal [19, 22–24]. Moreover, When Scherer, Shaffer [18] discuss why adding a detailed VAERS serious report to vaccine information decreased trust, their explanations point to the effect of two cognitive biases: confirmation bias [25] and availability bias [18]. They note that when people read the detailed report, it gives them the chance to see what they want to see, i.e., confirmation bias [25]. The other discussed possibility was that the detailed report increases the vividness of the AEs for the participants, i.e., availability bias [18]. Vaccination decision-making due to its associated factors such as perceived risk, ambiguity, uncertainty, and loss is prone to several cognitive biases that may disrupt people’s logical reasoning and may not maximize their self-interests [26]. However, the role of cognitive biases in vaccination decision-making has not been studied comprehensively [27, 28].

We follow two objectives in this paper. First, to address concerns regarding vaccination AEs, we will analyze reported AEs in VAERS from 2011 to 2018. Given that the summary information from VAERS has a potential role in increasing vaccine acceptance, we will provide a communicable summary of the VAERS database. To this aim, we measure the reporting rate, analyze the AEFI reports based on sex and age groups, and provide analyses of the most frequent vaccine groups and AEs. Then, we will focus on the Serious Adverse Event (SAE) reports by performing similar analyses. As our second objective, in this paper, we highlight the critical role of cognitive biases on vaccine hesitancy by identifying and classifying the potential cognitive biases that influence individuals’ vaccination decision-making. Providing a communicable summary of VAERS to be used in vaccine communication without mentioning the critical role of cognitive biases in vaccine decision-making would not be optimal. To this aim, we identify the potential cognitive biases affecting vaccine information communication, in particular, and vaccine hesitancy in general. We will also categorize them based on the mechanism that they get triggered in the vaccine decision-making process and discuss how they contribute to the vaccine hesitancy problem.

The contributions of this paper are two-fold. First, the analysis of the reported AEFIs will allow us to address vaccine concerns and provide empirical evidence regarding the severity of their AEs. Second, referring to the well-documented cognitive processes, we will investigate the relevance of the cognitive biases affecting vaccine-related decision-making in people concerned with vaccines’ confidence and risks.

## Method

We quantitatively analyzed AEFI reports in VAERS from 2011 to 2018. VAERS [29] is the primary system for collecting AEFIs to detect possible safety problems in U.S. licensed vaccines. VAERS is a passive spontaneous AEFI reporting system for vaccine safety monitoring whose primary purpose is safety signal detection and hypothesis generation about AEFIs [30]. AEFI is “any untoward medical occurrence which follows immunization and which does not necessarily have a causal relationship with the usage of the vaccine” [31]. VAERS, as a passive surveillance system, is subject to limitations such as underreporting, overreporting, missing information in fields, and lacking information on other causes of the reports or underlying medical conditions [32, 33]. However, VAERS is open to everyone to report voluntarily any medical incident after immunization, even if the reporter is not sure if the vaccine caused the incident [30]. It makes VAERS a rich data source for vaccine information communication to provide evidence-based trustful information about vaccine safety and AEs.

We collected all AEFI reports in VAERS from 2011 to 2018, excluding the U.S. territories and possessions data. Each report is for one patient who has taken one or more vaccines and has one or more AEs. Each report in VAERS is identified as SAE if it is life-threatening, results in death or disability, or requires hospitalization or prolongation of hospitalization. AEs recorded in VAERS are coded in the international Medical Dictionary for Regulatory Activities (MedDRA) Preferred Terms (PTs). PTs in VAERS are from versions 13.1 to 22.0. To provide a consistent view and accurate result, we converted all PTs from all other versions to PTs version 22.0. Following the age classification in [15], age groups are defined as < 1, 1–6, 7–17, 18–64, ≥65. Age groups also have the unknown group that refers to the AEFI reports with the missing age information.

The numbers of AEFI reports were calculated by year. The average reporting rates (AEFI and SAE reports per 1 million population) were calculated by dividing the average of reports over the 8 years by the average of the U.S. states population from 2011 to 2018 extracted from the U.S. census bureau. The average numbers of reports per year were calculated by dividing the number of reports per year by the total U.S. states population per year.

An AEFI report can have more than one vaccine type and AE. Frequently reported vaccine types and vaccine type combinations were defined as vaccine types and vaccine types combinations that had more than 100 reports. Frequently reported AEs were defined as AEs that were reported in more than 1000 reports.

## Results^1^

### Number of reports

**Fig. 1:**
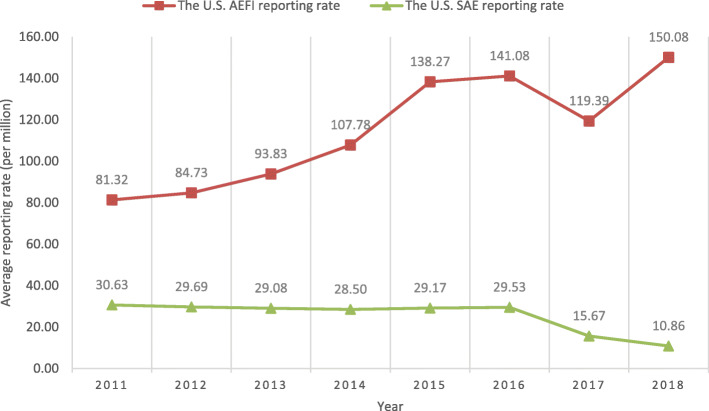
Population-based AEFI and SAE reporting rate in the U.S

From January 1, 2011, to December 31, 2018, VAERS received 293,609 reports (Supplementary Material 1, Table 1). In the 8 years, the average population-based reporting rate in the U.S. is 114.92 reports per 1 million population with an upward trend (Fig. 1). The percentage of children aged < 7 years is 15.7% (Supplementary Material 1, Table 2). It is the primary age group in the recommended vaccination schedules [15]. The 18–64 age group has the highest percentage of reports (31.23%). 51.8% of the patients are females (Supplementary Material 1, Table 3 and Table 4), while the males’ percentage is 27.2%. The sex of the rest of the reports is unknown (21%).

### Vaccine types

VAERS reports involve 87 different vaccine types, of which there could be different brands (205 different vaccine brands). There are mentions of 1 to 10 different vaccine types in each report. 78.2% of reports involve one, 12% involve two, 5.3% involve three, 3.4% involve four, and 1.11% involve 5 to 10 vaccine types. The 87 vaccine types and their combinations entail 5110 different vaccine combinations, of which 152 frequently reported vaccine types or vaccine type combinations (≥100 reports) comprise 91.8% of all reports (Supplementary Material 1, Table 5). The top five frequently reported vaccine types are Varicella-Zoster Vaccine (VARZOS: 14.3%), Influenza Virus Vaccine, Trivalent (FLU3: 11.53%), Pneumococcal Vaccine, Polyvalent (PPV: 6.18%), Human Papillomavirus 4-Valent (HPV4: 4.60%), and Influenza Virus Vaccine, Quadrivalent (FLU4: 4.57%).

VARZOS (14.27%) is the most frequently reported vaccine type, mainly due to the significant rise in its reports in 2018. In VAERS, the average number of VARZOS reports per year from 2011 to 2017 was 3502, whereas it increased almost five times to 17,395 in 2018. This huge rise lies in releasing a new vaccine brand (Shingrix) in the VARZOS group in 2017. Shingrix-related reports made up 79.6% of the VARZOS AEFIs and 30.2% of all AEFIs in 2018. If we remove Shingrix-related reports, the population-based AEFI reporting rate (Fig. 1) will have a downward trend from 2016. The significant number of reports for the new-released vaccine (Shingrix) could be explained by the Weber effect [34], an epidemiologic phenomenon that states the number of AEs for a new drug increases to its peak until the middle to end of the second year of marketing of that drug. The second most frequent vaccine type is FLU3 (11.53%), a type of Influenza vaccine. Considering all 13 Influenza vaccine types, 11 of them are among the high frequently reported vaccine types, making up 21.16% of all AEFIs.

### Adverse events

In 293,609 VAERS reports, 1,002,037 AEs were reported. Sixty thousand forty of the AEs are “No adverse event,” and 75,155 of them belong to “Medication errors and other product use errors and issues” as High-Level Group Term. In our analysis of the reports, we excluded these two groups as they are not considered AEs. This resulted in 225,046 reports involving 866,842 instances of AEs from 7092 different AEs. The range of AEs per report was from 1 to 155 (only one report). Removing the outliers (AEs per report: > 9), on average, each report involves 3.35 AEs, and the median is 3 (20.17%). The top 10 highly frequent AEs are: injection site erythema (4.29%), pyrexia (3.66%), injection site swelling (3.21%), injection site pain (3.2%), pain (2.78%), erythema (2.56%), pain in extremity (2.33%), headache (2.05%), injection site warmth (1.82%), and rash (1.8%). The highly frequent AEs in VAERS (≥1000) are reported in Supplementary Material 1, Table 6.

### Serious adverse events (SAE) reports

Out of the 293,609 VAERS reports, 16,130 (5.5%) were SAEs. In the 8 years, the U.S.’s average population-based SAE reporting rate is 25.3 reports per 1 million population (Fig. 1). Of the five reasons for SAE reports in VAERS, hospitalization (70%) is the main reason (Supplementary Material 1, Table 7). Next are disability (23.67%) and life-threatening (20.3%). In 7.46% of the SAE reports, the reason was death. In VAERS SAE reports, Influenza Virus Vaccine, Trivalent (FLU3: 15%) is the highest reported vaccine type (Supplementary Material 1, Table 8). Varicella-Zoster Vaccine (VARZOS: 10.75%), Pneumococcal Vaccine, Polyvalent (PPV: 5.56%), Human Papillomavirus 4-Valent (HPV4: 5.1%), and Influenza Virus Vaccine, Quadrivalent (FLU4: 4.87%) are next. The top five AEs reported in SAE reports are pyrexia (2.44%), pain (1,23%), vomiting (1.17%), headache (1.11%), and dyspnoea (1.05%) (Supplementary Material 1, Table 9). 33% of the patients reported with SAE ultimately recovered, 42.8% did not recover at the time of reporting, 21.4% were Unknown at the time of reporting, and for 3%, no information was reported about the recovering stage.

## Discussion

This study provided an aggregated summary of the VAERS data from 2011 to 2018 using the critical points of analysis for vaccine safety, including population-based AEFI report, vaccine-type, AEs, SAEs, and AEs and vaccine types in SAE reports. Also, we provided an interactive BI dashboard for healthcare professionals to use in vaccine communications with vaccine decision-makers (patients/parents). A link to this dashboard is provided in the Availability of data and materials section. Healthcare professionals can use this dashboard to communicate vaccine safety information at the general level and drill down to any specific vaccine type or AE to provide, for example, vaccine-specific or AE-specific information. Our results show that the population-based AEFI reporting in the U.S. has an upward trend. However, if we remove the Shingrix vaccine from the data, basing on the Weber effect [34], the AEFI reporting rate would have a downward trend from 2016 to 2018. In total, the average population-based report in the U.S. from 2011 to 2018 is 114.87 reports per 1 million population. Also, our results show that the highest frequent AEs were mild reactions such as injection site reaction, pain, headache, rash, fever, erythema, and chills that are expected from vaccination. We applied the same analysis on the top-10 highly frequent vaccine types and got the same results.

Overall, the VAERS database analysis showed that 94.5% of the received AEFIs are not serious. Moreover, an AEFI report only specifies a temporal association between immunization and the following AE. For an AEFI, suspicion alone is enough to report, and the reporter is not expected to assess the causality. The event’s cause could be due to the vaccine’s inherent properties, quality defects of the product, including the administration device, inappropriate vaccine handling, a cause unrelated to the vaccine, immunization error, or immunization anxiety [31]. VAERS staff also conduct further investigations and follow up on all SAE reports [35], “while these events (SAEs) can happen after vaccination, they are rarely caused by the vaccine” [36]. We also applied the same analysis as VAERS on Canada Vigilance (CV) [37], which is another publicly available AE data source at the country level (Supplementary Material 2). Results from CV and VAERS are consistent in terms of the average population-based report, age and sex distribution of the patients, vaccine combinations, and AEs. The highest frequent AEs were mild reactions such as injection site reaction, pain, headache, rash, and fever that are excepted from vaccination.

The result provided in this paper is built upon the recommendations by Harrison and Wu [13] and Blaisdell, Gutheil [14] to reconstruct vaccine safety communication by providing meaningful evidence-based information to the public. It follows the findings of Scherer, Shaffer [16] that the best form for communicating vaccine safety information is providing it in a summarized format. As trusted key elements in transmitting vaccine information [14, 17, 23–26], health practitioners can use the findings of this study to have open communication with vaccine decision-makers (patients/parents). Having access to a summary of VAERS data as evidence-based vaccine AEs information can help mitigating vaccine hesitancy concerns over vaccine safety and AEs, increase trust in vaccines, and eventually increase vaccine acceptance. Putting the result of this paper next to vaccine safety statements from trusted sources like CDC and other vaccine information like the total number of the population vaccinated or the number of vaccine doses administrated each year in the U.S. will have the highest effect.

This communication, however, is not free from challenges. One challenge, particularly when the information is about vaccine safety and AEs, is the role of cognitive biases that has not seriously been taken into consideration. At the individual level, people decide about vaccination based on several factors, including but not limited to their prior beliefs, perceived risk, perceived ambiguity, perceived loss, the message they receive, and the factors associated with it like the emotion and vividness of the message. All these factors instigate cognitive biases that might nudge people toward sub-optimal decisions. As Dubov and Phung [22] put it, people “make suboptimal decisions not because they lack information, but because of predictably irrational biases and cognitive errors.” People follow alternative forms of rationality and make decisions due to cognitive biases that do not necessarily maximize an individual’s self-interest [26].

Reconstructing vaccine communication to increase vaccine trust would not be successful unless we take cognitive biases that affect vaccine decision-making into consideration. Otherwise, the new forms of communication would still be affected by people’s cognitive biases. Not only will they not achieve a higher vaccine trust, but contrarily, they might backfire and cause distrust in vaccination. Cognitive biases affect people’s logical reasoning in different stages and aspects, and vaccine decision-making is not an exception. For example, cognitive biases come in different decision-making stages. Some affect an individual’s decision-making, and some others might nudge people to affirm themselves in the might-be-wrong decision they already have taken. Moreover, it is not only vaccine takers who might be affected by cognitive biases. Clinicians and other healthcare professionals are also influenced by these biases [38–40], affecting their vaccine recommendation. We have identified the most potential cognitive biases affecting vaccine communication and decision-making by reviewing the literature on cognitive biases and vaccine hesitancy. A summary of the discussed cognitive biases is provided in Table 1.

**Table 1:** Potential cognitive biases that contribute to vaccine hesitancy

Category	Cognitive Bias	Definition	Example
**Group 1**: Cognitive biases triggered by processing vaccine-related information	Framing effect	Formulating a message with no change in the main content will affect the agent’s choice [41].	Negatively framing the outcomes of a vaccine by emphasizing the smaller portion of patients with AEs than most patients with no AEs.
	Base rate neglect	The tendency to focus on specific information and ignore general information even though the general information is more important [42].	Overestimating rare AEFIs and underestimating common mild AEFIs [43].
	Availability bias	The tendency to attribute higher weight to factors that are easier to recall [18].	A rare SAE report’s media coverage offers a vivid and emotionally compelling message, likely to be recalled during vaccination decision making [22].
	Anchoring effect	The tendency to rely heavily on an initially presented value when making a decision [17].	Seeing an SAE following a vaccine and believe SAEs are more common with that specific vaccine [44].
	Authority bias	The tendency to attribute more weight to the opinion of authoritative figures [45].	As an authoritative figure, when a medical professional spreads anti-vaccination content, it could instigate people to opt against vaccination.
**Group 2**: Cognitive biases triggered in vaccination decision making	Omission bias	The tendency to consider the outcomes of not doing an action (omission) as less severe than doing the action (commission), even if the result of not doing is more severe than or equal to doing the action [19].	Parents consider vaccination as commission, and when they anticipate AEFIs, they tend to omission (not vaccinating).
	Ambiguity aversion	The tendency to take a known risk over the unknown risk, regardless of the outcomes [21].	People prefer a known risk from a disease rather than a more ambiguous risk of a vaccine for the same disease [23].
	Loss aversion	The tendency to put greater weight on avoiding losses than achieving comparable gains [20].	When describing AEFIs, patients may only focus on a 1% chance of having AEs instead of a 99% chance of no AEs [24].
	Optimism bias	The tendency to have an unrealistically optimistic view about a particular health risk, believing it is higher for other people than oneself [22].	People do not consider themselves at risk from flu, assuming themselves as healthy, not susceptible to flu, and strong enough to fight [46].
	Present bias	The tendency to put more weight on the costs and benefits today and less weight on those realized in the future [22].	Vaccine AEs (as a cost) are more visible to people, so they receive more weight. Immunity to a disease as a future benefit is not visible and receives less weight.
	Protected values	The tendency to protect absolute and not amenable-to-intervention values that people think should not be traded off [47].	Believing in parents’ right to refuse vaccination [38].
**Group 3**: Cognitive biases triggered by prior beliefs regarding vaccination	Confirmation bias	The tendency to recall and interpret information that confirms our existing beliefs [25].	Vaccine-hesitant people consider a vaccine-preventable disease as less dangerous and overestimate AEFIs [26, 48].
	Belief bias	The tendency to evaluate an argument’s validity based on the believability of the conclusion [49].	Discussing vaccine safety in terms of mild AEFIs with individuals who believe vaccination policies are motivated by big corporations’ profit would be ineffective.
	Shared information bias	The tendency to spend more time and energy on the information that members of a group are familiar with and less time and energy on new information [50].	Focusing on a limited number of anti-vaccine topics like the debunked MMR-autism link on online anti-vaccine echo-chambers.
	False consensus effect	The tendency to overestimate the extent to which the general population share one’s belief [51].	On social media, vaccine-hesitant (vaccine-advocate) mothers are more (less) likely to engage in communication about the issue [52]. It creates online communities with high false consensus on vaccine-hesitancy.

To identify cognitive biases affecting vaccine hesitancy, we first searched for the peer-reviewed articles on SCOPUS and Web of Science databases that contain the term *vaccine* plus *cognitive bias* or *heuristic* in their title, abstract, or keywords. A total of 196 articles were obtained that were reduced to 132 records after removing the duplicates. Next, we included the 22 articles relevant to cognitive biases’ effects on vaccine hesitancy during the screening process and excluded all the others. Finally, we added seven more articles by searching the references of the included articles. Reviewing the 29 articles resulted in 13 cognitive biases. As there was no work analyzing the effect of cognitive biases on vaccine hesitancy in social media, we conducted separate research on the topic of prevalent cognitive biases on social media. We found two cognitive biases, shared information bias and false consensus effect, that can affect and direct social media users’ behaviors. Since vaccine hesitancy is prevalent on social media, we also added these two biases to the list of cognitive biases affecting vaccine hesitancy (Table 1).

In compiling the list of cognitive biases that affect vaccine hesitancy, we categorized them into three categories: Group 1: Cognitive biases triggered by processing vaccine-related information; Group 2: Cognitive biases triggered in the vaccination decision making; and Group 3: Cognitive biases triggered by prior beliefs regarding vaccination. This categorization is based on the common factors for each group. Cognitive biases of Group 1, which are heavily dependent on the message, the content of the message, and the relevant factors such as its framing [41, 53] and emotions [54, 55], have a significant effect. The cognitive biases of Group 2 are triggered by factors that are most prominent when people are in the decision-making process. Vaccine decision-making is a process under uncertainty [23, 46, 48, 56] where the decision-maker’s risk perception plays the major role [19, 46, 57]. People’s capability to quantify the risk involved is limited in this setting, and they have ambiguity about the result [48, 56, 57]. Uncertainty, risk-perception, ambiguity, and other factors create a situation where the cognitive biases of Group 2 have the highest effect on people’s decisions. All cognitive biases in Group 3 have a common feature that is a prior-formed belief regarding vaccination. In this phase, people’s prior beliefs have a higher impact on their actions than the content of the message they receive. At this stage, decision-makers tend to keep their prior belief as the new contradictory information creates cognitive dissonance [58]. Another name for Group 3 would be cognitive biases that vaccine-hesitant people hold.

**Fig. 2:**
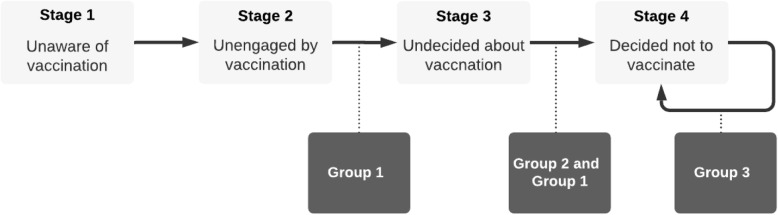
Mapping the three groups of vaccine hesitancy cognitive biases to the four stages of PAMP model

This categorization is also backed and guided by the three general phases of decision-making: pre-decision making, decision-making, post-decision making, and the Precaution Adoption Process Model (PAPM) [59]. PAPM is a stage-based theoretical model to explain how people decide to take a health-protective behavior and how they transform that decision to action through 7 stages: 1. unaware of the issue; 2. Unengaged by issue; 3. Undecided about acting; 4. Decided not to act; 5. Decided to act; 6. Acting; and 7. Maintenance. As this paper focuses on the vaccine hesitancy issue, which results in not vaccinating (Stage 4. Decided not to act), we explain the cognitive biases based on stages 1 to 4 (Fig. 2).

### Group 1: cognitive biases triggered by processing vaccine-related information

PAMP’s Stage 1 is when people have not heard about vaccination for a specific vaccine. From the very moment that people hear about vaccination, they are no more in Stage 1 and move to Stage 2, where they are aware of the vaccination, but they never consider taking any action about it. Stage 1 and 2 are in the pre-decision-making phase where the cognitive biases of Group 1 (message-dependent cognitive biases) have the only effect. In stage 1 or 2, vaccine decision-makers have no formed beliefs regarding the specific vaccination. Moreover, as they are not in the decision-making process yet (Stage 3), factors like uncertainty, risk, and ambiguity do not play major roles. In such settings, the message’s content and its underlying factors have the highest effect on people. These underlying factors, such as how the information is framed, its source, its level of emotion, and other factors trigger framing effect, availability bias, anchoring effect, base rate neglect, and authority bias. These cognitive biases can move people from Stage 2 to Stage 3 with a tendency toward vaccine hesitancy, or the effect might be strong enough to move vaccine decision-makers from Stage 3 to Stage 4 (Fig. 2).

We did not add the transition from Stage 1 to Stage 2 as the main distinction between these two is only whether the decision-maker knows about vaccination or not. However, in both, the individual has never thought about the vaccination. So, it is not a cognitive bias that moves decision-makers from Stage 1 to Stage 2, but the message itself makes decision-makers aware of the vaccination [59]. The same rule applies to moving from Stage 2 to Stage 3. In a compelling form, the message moves people from Stage 2 to Stage 3, where they start considering the vaccination, but there are undecided about it [59]. However, in this transition, message-related cognitive biases (Group 1) might create a tendency in decision-makers toward vaccine hesitancy. The other place for Group 1 cognitive biases’ effect is moving decision-makers from Stage 3 to Stage 4. As Weinstein, Sandman [59] suggest, the media (message) has the highest effect in moving people from Stage 1 to Stage 2, Stage 2 to Stage 3, and it has much less influence after that. Forming an opinion (Stage 3 to Stage 4) tends to be more influenced by individual sorts of factors than the message itself [59]. Here comes the main effect of Group 1 and Group 2 cognitive biases (Fig. 2). If the Group 1 cognitive biases’ influence is strong enough, they might nudge decision-makers to decide not to take vaccine (Moving from Stage 3 to Stage 4). The effect of Group 2 cognitive biases will be discussed below.

Framing effect happens when the formulation of a message, with no change in the main content, affects the agent’s choice [41]. In Tversky and Kahneman [41], participants were more willing to choose a program narrated in a risk-averse frame to combat a hypothetical disease rather than a risk-taking frame, though the outcomes were the same. The literature has emphasized the role of the framing effect to increase vaccine acceptance by positively narrating messages [22, 23, 60]. However, the reverse is also true as anti-vaxxers negatively frame vaccine-related information and vaccination outcomes that entails vaccine hesitancy.

Base rate neglect is the tendency to focus on specific information and ignore general information, even though the general information is more important [42]. Overestimating rare AEFIs and underestimating common mild AEFIs is an example [43]. Although the probability of a rare AEFI is much less than common mild AEFIs, people tend to ignore denominators in their vaccine risk perceptions. Base rate neglect, resulting from the difficulty that people have with ratio concepts [61] has the main effect when two sides of an argument are in the same context. However, when a single anecdote of an SAE outweighs the majority of mild AEs, other factors come into play, triggering other cognitive biases. For example, a personal narrative of one’s child suffering from an AEFI is significantly more powerful than the majority of mild AEs, regardless of its low probability [62, 63]. In such cases, emotions also come into play, providing a vivid narrative for decision-makers, a phenomenon called availability bias.

Availability bias is the tendency to attribute higher weight to factors that are easier to recall [18]. Media coverage of a rare SAE report that offers a vivid and emotionally compelling anti-vaccination message, likely to be recalled during decision making [22], could cause people to overestimate the probability of an AEFI [64]. The same rule applies to detailed reports in VAERS. In addition to the patient, vaccine, and AE information, an AEFI report includes an AE description that might increase the vividness and availability of that AE to the reader. This justifies findings by Scherer, Shaffer [16] that found VAERS detailed reported decreased people’s vaccine trust and acceptance.

Anchoring effect (first-impression bias) is the tendency to rely heavily on an initially presented value when making a decision [17]. In offering the HPV vaccine to patients, clinicians would be influenced by the age or a specific physical characteristic like a patient’s pubertal status [38]. Such first impressions would impair further cognitive processing of people and nudge them towards vaccine hesitancy.

Authority bias is the tendency to attribute more weight to the opinion of authoritative figures [45]. It could be used either in favor or against vaccination, depending on the authoritative figures’ viewpoint. Medical professionals are reliable sources of vaccine information, hence authoritative figures in this context. They generally support vaccination, and their authority contributes in favor of vaccination. However, when an authoritative figure spreads anti-vaccination content, it could instigate people to opt against vaccination [65].

### Group 2: cognitive biases triggered in vaccination decision-making

Group 2 cognitive biases are related to Stage 3 when people are in the vaccination decision-making (Fig. 2). This process is under uncertainty [23, 46, 48, 56], involves risk perception and assessment [19, 46, 57], and the result is unknown to some degree [46, 48, 56, 57] which makes it ambiguous for the decision-makers. Under such conditions, people use heuristics to simplify complex tasks to simpler judgmental operations [17]. Consequently, using these heuristics in imperfect conditions leads to cognitive biases. In vaccine decision-making, we have identified four cognitive biases instigated in this setting: omission bias, ambiguity aversion, loss aversion, and optimism bias. Present bias and protected values are two other biases that we identified belonging to Group 2 as they are mainly triggered in the decision-making phase (Stage 3). However, they have other triggers than risk perception and ambiguity. In present bias, time perception is the main factor as subjects tend to put more weight on immediate costs than delayed rewards [66]. In protected values, a specific belief is protected against any intervention no matter the consequences [67].

As mentioned earlier, Group 1 cognitive biases also contribute to moving from Stage 3 to Stage 4. Their effect might be strong enough for moving from Stage 3 to Stage 4, or they might exacerbate the effect of Group 2 cognitive biases as the effect of availability bias (from Group 1) on omission bias (from Group 2). Another case is also possible when a cognitive bias from Group 2 (e.g., ambiguity aversion or loss aversion) contributes to another cognitive bias in Group 2 (e.g., omission bias).

Omission bias is the tendency to consider the outcomes of not doing an action (omission) as less severe than doing it (commission), even if the result of omission is more severe than or equal to commission [19]. Investigating the role of omission bias in parents’ vaccine hesitancy, Ritov and Baron [57] revealed that parents have a strong tendency to omission when vaccinating might cause AEs. Parents consider vaccination’s side effects as being significantly worse than the same side effects from a disease in severity and duration [43]. The strong tendency to omission in vaccination decision-making lies in such factors as anticipated responsibility and regret [54]. Moreover, Availability bias exacerbates omission bias as the adverse outcomes of a decision become more available to the decision-makers [43]. When imperfect information about SAE reports becomes more available to vaccine-hesitant people, availability bias may force the decision-makers to omission. Contrarily, researchers suggest vaccination can be framed as omission and not vaccinating as commission. By doing so, omission bias could entail vaccine acceptance [19, 54].

Ambiguity aversion is the tendency to take a known risk over an unknown risk, regardless of the outcomes [21]. Ambiguity aversion as a potential reason for vaccine hesitancy [22, 26, 60, 68] happens when people prefer a known risk from a disease rather than a more ambiguous risk of a vaccine for that disease [23]. Ambiguity, by itself, also intensifies omission bias [43]. When the ambiguity about the outcome of vaccination increases, decision-makers have a greater tendency to omission rather than commission.

Loss aversion is the tendency to put greater weight on avoiding losses than achieving comparable gains [20]. When describing AEFIs, patients may only focus on a 1% chance of having AEs instead of a 99% chance of no AEs [24]. The same rule applies when people evaluate vaccination outcomes in the forms of omission (not vaccinating) and commission (vaccinating). The loss aversion from commission is higher than the loss aversion from omission [68].

Optimism bias is the tendency to have an unrealistically optimistic view about a particular health risk, believing it is higher for other people than oneself [22]. It is equivalent to assuming the probability of getting a Vaccine-Preventable Disease (VPD) is less for oneself than for other people. For example, optimism bias causes people not to consider themselves at risk from flu, assuming themselves as healthy, not susceptible to flu, and strong enough to fight [46]. Another example is clinicians’ optimism about their patients’ low risk of HPV acquisition, only because of their longstanding relationships with patients and their families [38].

Present bias is the tendency to put more weight on the costs and benefits today and less weight on those realized in the future [22]. In vaccination, vaccine AEs and costs are visible to people, so individuals put more weight on them as they are present to the decision-makers. However, potential future benefits (immunity to VPD) are less visible, so they receive less weight.

Protected values are absolute and not amenable to intervention values that people resist tradeoff against those regardless of the consequences [38, 47]. In vaccine decision-making, protected values are any beliefs that vaccination is in contrast with, and consequences are the harms that might arise from not vaccinating. No matter how small the sacrifice, how large the benefits, or how severe the consequences, the subjects do not trade-off those values [67]. Protected values could be people’s or children’s death [57] even if it is rare to happen, no necessity of HPV vaccination for boys, or parents’ right to refuse vaccination [38]. All these cases result in Stage 4: decided not to vaccinate. Protected values also have been used to explain omission bias as it increases people’s tendency to omission, even if its outcome is more harmful than commission [57].

### Group 3: cognitive biases triggered by prior beliefs regarding vaccination or cognitive biases that vaccine-hesitant people hold

At Stage 4, when people have a prior belief about a topic, their formed opinion (decided not to vaccinate) will affect their reaction to new information and arguments. They show different responses to information compared to Stage 3 and are more resistant to persuasion [59]. At Stage 4, people tend to read supporting information as opposed to conflicting information (confirmation bias) [69]. People’s assessment of the logical validity of an argument’s conclusion is affected by their believability in that conclusion (belief bias) [49]. On social media, people’s behavior affected by these biases might create polarized groups of like-minded people, i.e., echo chambers [70], where they are at risk of other social-level biases. In echo-chambers, people spend most of their time on already known and shared content (shared information bias) [50], which causes them to overestimate the extent to which the general population share one’s beliefs [51]. Cognitive biases of Group 3 have the highest effect at stage 4 that keeps vaccine-hesitant people hard to persuade and keep their already formed decision (not to vaccinate).

Confirmation bias is the tendency to information that confirms our existing beliefs [25]. It thwarts attempts to discredit false information that vaccine-hesitant people hold, as they tend to ignore evidence contrary to their beliefs [71]. It causes vaccine-hesitant people to consider a VPD less dangerous and overestimate AEFIs [26, 48].

Belief bias is the tendency to evaluate an argument’s validity based on the conclusion’s believability [49]. It thwarts individuals’ cognitive abilities in interpreting new evidence if it contradicts previously held beliefs. Anti-vaxxers’ arguments are in a wide range from the safety and effectiveness of vaccines, alternative medicine, civil liberties, conspiracy theories, morality, religion, and ideology [8]. After being exposed to and believing in such content, confirmation bias would prevent people from paying attention to new contradictory information. Even after engaging with such content, belief bias would impede individuals’ cognitive abilities to verify new information’s validity.

Shared information bias is the tendency to spend more time and energy on the information that members of a group are familiar with and less time and energy on new information [50]. On social media, users in anti-vaccination groups share vaccine misinformation, and the following interactions and discussions are around those topics. In such groups, members could depict biases like confirmation and belief bias when encountering and interpreting new information. It entails shared information bias, which in turn might increase the false consensus effect.

False consensus effect is the tendency to overestimate the extent to which the general population share one’s belief (judging one’s beliefs to be more common than they actually are) [51]. On social media, vaccine-hesitant mothers are more likely to engage in communication about the issue. In contrast, mothers supporting vaccination are less likely to engage in such communications [52]. These two groups create online communities with high false consensus on vaccine-hesitancy concerns; small yet powerful online anti-vaccination communities that result in behaviors and attitudes favoring anti-vaccination beliefs [52, 72].

In addition to the cognitive bias that might contribute to vaccine hesitancy, other cognitive biases like default and bandwagon effect can help explain vaccine hesitancy, depending on how we frame them. The default effect is the tendency to go with the default choice when we have to choose between several options [73]. By making vaccination the default choice, more people tend to choose it (vaccination) [19, 60]. The bandwagon effect is the tendency to make a decision based on the decision of the majority of other people [74]. Described as “jumping on the bandwagon” [74], it could be because people may think other people have already made a logical decision [74] or because of social pressure [75]. It is a significant motivator for vaccination decision-making, depending on how the information is framed [74]. Emphasizing the social pressure of vaccinating, for example, makes the bandwagon effect apparent. However, if the information emphasizes other aspects, like herd immunity, the bandwagon becomes ineffective.

The identified cognitive biases and the categorization can help public health officials in vaccine communication to increase vaccine trust and acceptance. Based on the categories provided in this paper, public health officials can customize their plans, interventions, and other forms of communications to hinder the impact of identified cognitive biases. First, they should consider Group 2 cognitive biases in all their plans. Public health officials should try to decrease perceived levels of uncertainty, ambiguity, and feeling of loss about the result of vaccination in vaccine decision-makers. The result of this paper, the summarized data of the VAERS AEFI reports, could be of help here to communicate evidence-based information about vaccine safety and AEs to decrease the influence of those factors.

Group 1 cognitive biases are important as both sides (anti-vaxxers and vaccine advocates) can use them. Public health officials should have campaigns to decrease the effect of these cognitive biases prevalent in anti-vaccine content. Such efforts could be formed to increase the availability of vaccine-advocate information and emphasizing the majority of mild AEFIs to reduce the effect of base rate neglect. More importantly, by using some of the biases like the framing effect and authority bias, public health officials could improve vaccine trust. For example, positive framing of vaccine side effects (high probability of no side effects) is more effective for low-involved women (women with no infant or intention of having a baby) [53]. On social media, the CDC’s correction of health-related misinformation proved to be effective [76], which could be an example of authority bias. Alleviating the effect of Group 3, cognitive biases that vaccine-hesitant people hold, need careful attention. One commonly agreed strategy is not direct debunking or refuting misinformation as this might backfire [77, 78]. It is because direct debunking requires repeating the misinformation. Repeating increases fluency (the ease of information recall) [77] of the misinformation that positively affects confirmation bias.

## Limitations and future work

In analyzing VAERS data, we tried to summarize it by focusing on the vaccine safety issue and providing tangible understandings of vaccine safety’s critical aspects. We followed the directions from past work [15] and consulted with the field experts. However, other analysis aspects might be useful for vaccine safety communication that are extractable from VAERS data, but we have not covered them, for example, analysis based on states. Furthermore, we did not have access to other sources related to vaccine safety, for example, the number of administered vaccine doses in the U.S. or the population who received vaccines. Combining this paper’s result with the related information sources will increase the validity of the summarized data and make it more meaningful in vaccine information communication with vaccine decision-makers (patients/parents). For example, the number of AEFI reports per total administrated vaccine doses in the U.S. will give a better sense to the vaccine decision-makers about vaccine safety. Future work can analyze VAERS data from new aspects in vaccine safety, e.g., the geographical distribution of AEFIs. They could also combine VAERS data with other vaccine safety sources to provide a more comprehensive vaccine safety data analysis.

Although we tried to follow a systematic review’s guideline in identifying cognitive biases in vaccine hesitancy, the result could not be considered a systematic review primarily due to space limitations. Moreover, this paper’s focus was identifying all the potential cognitive biases affecting vaccine hesitancy and introducing them to the researchers and practitioners in the field based on a grounded theoretical approach. However, we did not cover several essential aspects, such as the suggested interventions for the cognitive biases, causing factors for each cognitive biases and their mechanism of effect, or the detailed mechanism of movement between stages caused by the cognitive biases. Future work can extend the current study by systematically reviewing the cognitive biases in the vaccine hesitancy domain. It can also extend to aspects not mentioned in our work, including but not limited to suggested interventions and causing factors for the cognitive biases, movement mechanisms between stages, or using other grounded health-behavior models.

## Conclusions

Vaccine hesitancy is a recent public health challenge that became especially critical in the ongoing COVID-19 pandemic. This paper provided public health practitioners and researchers with data-driven evidence-based information supporting vaccines’ safety concerning potential AEs. The paper helps provide new forms of vaccine safety communications to increase vaccine trust and acceptance. Furthermore, we have identified and analyzed the potential cognitive biases that affect vaccine communication and decision-making and cause vaccine hesitancy. Public health officials could consider the effect of these cognitive biases in any plans, interventions, or other vaccine communications and modify them to increase vaccine trust and acceptability, particularly for the COVID-19 vaccines.

## Supplementary Information

**Additional file 1: Table S1.** The annual number of AEFI reports for VAERS, 2011–2018. **Table S2.** VAERS AEFI reports by age groups, 2011–2018. **Table S3.** VAERS AEFI reports by sex groups, 2011–2018. **Table S4.** Sex-Age distribution in VAERS AEFI reports. **Table S5.** VAERS AEFI reports by vaccine combinations, 2011–2018. **Table S6.** The highly frequent AEs in VAERS, 2011–2018. **Table S7.** VAERS SAE report categories, 2011–2018. **Table S8.** VAERS SAE reports by vaccine combinations, 2011–2018. **Table S9.** The highly frequent AEs for SAEs in VAERS, 2011–2018.

**Additional file 2: Supplementary Material 2.** Canada Vigilance database results.

## Data Availability

The datasets generated and/or analysed during the current study are available in the summarized VAERS Reports repository, https://public.tableau.com/profile/aefi#!/vizhome/VAERSAdverseEventFollowingImmuinzationAEFIReports2011-2018/Dashboard1.

## Abbreviations

AE: Adverse Event
AEFI: Adverse events following immunization
MedDRA: Medical Dictionary for Regulatory Activities
PAPM: Precaution Adoption Process Model
PT: Preferred Term
SAE: Serious Adverse Event
SAGE: Strategic Advisory Group of Experts
VAERS: Vaccine Adverse Event Reporting System
VPD: Vaccine-Preventable Disease

## Footnotes

1 All the results mentioned here, in addition to more data, are published in the form of an interactive BI dashboard: A link to this dashboard is available in the Availability of data and materials section.
