# Supplementary material for: Vaccine hesitancy: evidence from an adverse events following immunization database, and the role of cognitive biases
Source: BMC Public Health. 2021 Sep 16;21:1686. doi: 10.1186/s12889-021-11745-1 (PMC8444164; doi:10.1186/s12889-021-11745-1)
Supplement: Supplementary file 1 — Additional file 1: Table S1. The annual number of AEFI reports for VAERS, 2011–2018. Table S2. VAERS AEFI reports by age groups, 2011–2018. Table S3. VAERS AEFI reports by sex groups, 2011–2018. Table S4. Sex-Age distribution in VAERS AEFI reports. Table S5. VAERS AEFI reports by vaccine combinations, 2011–2018. Table S6. The highly frequent AEs in VAERS, 2011–2018. Table S7. VAERS SAE report categories, 2011–2018. Table S8. VAERS SAE reports by vaccine combinations, 2011–2018. Table S9. The highly frequent AEs for SAEs in VAERS, 2011–2018. [file 12889_2021_11745_MOESM1_ESM.docx]

1. Tables and Figures

In addition to the supplementary material, results are published in the form of an interactive BI dashboard: [Link to VAERS AEFI reports 2011 - 2018](https://public.tableau.com/views/VAERSAdverseEventFollowingImmuinzationAEFIReports2011-2018/Dashboard1?:language=en&:display_count=y&:origin=viz_share_link)

Table 1. The annual number of AEFI reports for VAERS, 2011-2018

| Year | VAERS reports |
| --- | --- |
| 2011 | 25336 |
| 2012 | 26590 |
| 2013 | 29651 |
| 2014 | 34307 |
| 2015 | 44335 |
| 2016 | 45561 |
| 2017 | 38800 |
| 2018 | 49029 |
| Total | **293609** |

Table 2. VAERS AEFI reports by age groups, 2011-2018

| **Age** | | | | | | | | | | | | | | |
| --- | --- | --- | --- | --- | --- | --- | --- | --- | --- | --- | --- | --- | --- | --- |
|  | **<1** | | **1-6** | | **7-17** | | **18-64** | | **≥65** | | **Unknown** | | **Total** | |
| **Year** | **No.** | **(%)** | **No.** | **(%)** | **No.** | **(%)** | **No.** | **(%)** | **No.** | **(%)** | **No.** | **(%)** | **No.** | **(%)** |
| **2011** | 1792 | 7.07 | 4167 | 16.45 | 3982 | 15.72 | 9947 | 39.26 | 3666 | 14.47 | 1782 | 7.03 | **25336** | **100** |
| **2012** | 1734 | 6.52 | 3419 | 12.86 | 3387 | 12.74 | 10504 | 39.50 | 4476 | 16.83 | 3070 | 11.55 | **26590** | **100** |
| **2013** | 1593 | 5.37 | 3620 | 12.21 | 3230 | 10.89 | 11283 | 38.05 | 5023 | 16.94 | 4902 | 16.53 | **29651** | **100** |
| **2014** | 1886 | 5.50 | 3950 | 11.51 | 3822 | 11.14 | 11386 | 33.19 | 4995 | 14.56 | 8268 | 24.10 | **34307** | **100** |
| **2015** | 2122 | 4.79 | 4246 | 9.58 | 4300 | 9.70 | 11899 | 26.84 | 6869 | 15.49 | 14899 | 33.61 | **44335** | **100** |
| **2016** | 1911 | 4.19 | 3747 | 8.22 | 4842 | 10.63 | 11539 | 25.33 | 7006 | 15.38 | 16516 | 36.25 | **45561** | **100** |
| **2017** | 1933 | 4.98 | 4432 | 11.42 | 3866 | 9.96 | 10416 | 26.85 | 7165 | 18.47 | 10988 | 28.32 | **38800** | **100** |
| **2018** | 1663 | 3.39 | 3916 | 7.99 | 3703 | 7.55 | 14733 | 30.05 | 12345 | 25.18 | 12669 | 25.84 | **49029** | **100** |
| **Total** | **14634** | **4.98** | **31497** | **10.73** | **31132** | **10.60** | **91707** | **31.23** | **51545** | **17.56** | **73094** | **24.90** | **293609** | **100** |

Table 3. VAERS AEFI reports by sex groups, 2011-2018‎

|  | **SEX** | | | | | | | |
| --- | --- | --- | --- | --- | --- | --- | --- | --- |
|  | **Female** | | **Male** | | **Unknown** | | **Total** | |
|  | **No.** | **(%)** | **No.** | **(%)** | **No.** | **(%)** | **No.** | **(%)** |
| **2011** | 15,623 | 61.66 | 8,753 | 34.55 | 960 | 3.79 | **25,336** | **100** |
| **2012** | 16,121 | 60.63 | 8,514 | 32.02 | 1,955 | 7.35 | **26,590** | **100** |
| **2013** | 17,102 | 57.68 | 8,833 | 29.79 | 3,716 | 12.53 | **29,651** | **100** |
| **2014** | 17,219 | 50.19 | 9,150 | 26.67 | 7,938 | 23.14 | **34,307** | **100** |
| **2015** | 19,324 | 43.59 | 10,383 | 23.42 | 14,628 | 32.99 | **44,335** | **100** |
| **2016** | 19,403 | 42.59 | 10,410 | 22.85 | 15,748 | 34.56 | **45,561** | **100** |
| **2017** | 19,084 | 49.19 | 9,647 | 24.86 | 10,069 | 25.95 | **38,800** | **100** |
| **2018** | 28,243 | 57.60 | 14,139 | 28.84 | 6,647 | 13.56 | **49,029** | **100** |
| **Total** | **152,119** | **51.81** | **79,829** | **27.19** | **61,661** | **21.00** | **293,609** | **100** |

Table 4. Sex-Age distribution in VAERS AEFI reports

|  | **Age** | | | | | | | | | | | | | |
| --- | --- | --- | --- | --- | --- | --- | --- | --- | --- | --- | --- | --- | --- | --- |
|  | **<1** | | **1-6** | | **7-17** | | **18-64** | | **‎≥65‎** | | **Unknown** | | **Total** | |
| **SEX** | **No.** | **(%)** | **No.** | **(%)** | **No.** | **(%)** | **No.** | **(%)** | **No.** | **(%)** | **No.** | **(%)** | **No.** | **(%)** |
| **Female** | 5864 | 2.00 | 13236 | 4.51 | 15831 | 5.39 | 66058 | 22.50 | 36103 | 12.30 | 15027 | 5.12 | **152119** | **51.81** |
| **Male** | 6578 | 2.24 | 14787 | 5.04 | 13013 | 4.43 | 23570 | 8.03 | 14397 | 4.90 | 7484 | 2.55 | **79829** | **27.19** |
| **Unknown** | 2192 | 0.75 | 3474 | 1.18 | 2288 | 0.78 | 2079 | 0.71 | 1045 | 0.36 | 50583 | 17.23 | **61661** | **21.00** |
| **Total** | **14634** | **4.98** | **31497** | **10.73** | **31132** | **10.60** | **91707** | **31.23** | **51545** | **17.56** | **73094** | **24.90** | **293609** | **100.00** |

Table 5. VAERS AEFI reports by vaccine combinations, 2011-2018^[[1]](#footnote-2)^

| **Vaccine Combination** | **No.** | **(%)** | **Total %** |
| --- | --- | --- | --- |
| VARZOS | 41912 | 14.27% | 14.27% |
| FLU3 | 33850 | 11.53% | 25.80% |
| PPV | 18144 | 6.18% | 31.98% |
| HPV4 | 13516 | 4.60% | 36.59% |
| FLU4 | 13415 | 4.57% | 41.16% |
| VARCEL | 13334 | 4.54% | 45.70% |
| TDAP | 9668 | 3.29% | 48.99% |
| PNC13 | 7930 | 2.70% | 51.69% |
| HEP | 7892 | 2.69% | 54.38% |
| MMR | 7773 | 2.65% | 57.03% |
| HPV9 | 7255 | 2.47% | 59.50% |
| FLUX | 6534 | 2.23% | 61.72% |
| RV5 | 6175 | 2.10% | 63.83% |
| HEPA | 5531 | 1.88% | 65.71% |
| FLU3+PPV | 5398 | 1.84% | 67.55% |
| MMRV | 5370 | 1.83% | 69.38% |
| MNQ | 3987 | 1.36% | 70.73% |
| HIBV | 2632 | 0.90% | 71.63% |
| DTAP | 2535 | 0.86% | 72.49% |
| MENB | 2436 | 0.83% | 73.32% |
| FLUN4 | 2400 | 0.82% | 74.14% |
| FLUN3 | 1718 | 0.59% | 74.73% |
| DTAPIPV+MMRV | 1705 | 0.58% | 75.31% |
| FLUC4 | 1697 | 0.58% | 75.89% |
| MNQ+TDAP | 1455 | 0.50% | 76.38% |
| MMR+VARCEL | 1364 | 0.46% | 76.85% |
| FLU3+PNC13 | 1313 | 0.45% | 77.29% |
| FLU4+PPV | 1224 | 0.42% | 77.71% |
| DTAPIPV | 1095 | 0.37% | 78.08% |
| UNK | 1086 | 0.37% | 78.45% |
| DTAPHEPBIP+HIBV+PNC13+RV5 | 1055 | 0.36% | 78.81% |
| DTAPIPV+MMR+VARCEL | 960 | 0.33% | 79.14% |
| FLUA3 | 929 | 0.32% | 79.46% |
| FLU3+VARZOS | 925 | 0.32% | 79.77% |
| ANTH | 915 | 0.31% | 80.08% |
| DTAPIPVHIB+PNC13+RV5 | 912 | 0.31% | 80.39% |
| FLUC3 | 911 | 0.31% | 80.70% |
| HEPA+MMR+VARCEL | 853 | 0.29% | 80.99% |
| DTAPIPVHIB | 816 | 0.28% | 81.27% |
| HPV4+MNQ+TDAP | 801 | 0.27% | 81.54% |
| SMALL | 786 | 0.27% | 81.81% |
| RAB | 781 | 0.27% | 82.08% |
| PPV+TDAP | 779 | 0.27% | 82.34% |
| TYP | 771 | 0.26% | 82.61% |
| DTAP+IPV+MMR+VARCEL | 767 | 0.26% | 82.87% |
| DTAPHEPBIP+HIBV+PNC13+RV1 | 757 | 0.26% | 83.12% |
| TD | 725 | 0.25% | 83.37% |
| FLUX+PPV | 721 | 0.25% | 83.62% |
| FLU3+TDAP | 688 | 0.23% | 83.85% |
| DTAPIPVHIB+HEP+PNC13+RV5 | 665 | 0.23% | 84.08% |
| HPV4+MNQ | 580 | 0.20% | 84.28% |
| HPVX | 577 | 0.20% | 84.47% |
| MMRV+VARCEL | 570 | 0.19% | 84.67% |
| HPV9+MNQ+TDAP | 570 | 0.19% | 84.86% |
| HEPAB | 557 | 0.19% | 85.05% |
| PPV+VARZOS | 530 | 0.18% | 85.23% |
| MENB+MNQ | 513 | 0.17% | 85.41% |
| YF | 503 | 0.17% | 85.58% |
| HEPA+MMR+PNC13+VARCEL | 430 | 0.15% | 85.72% |
| TDAP+VARZOS | 416 | 0.14% | 85.86% |
| TTOX | 391 | 0.13% | 86.00% |
| HEPA+HPV4 | 383 | 0.13% | 86.13% |
| MNQ+TDAP+VARCEL | 375 | 0.13% | 86.26% |
| DTAPHEPBIP+HIBV+PNC13 | 365 | 0.12% | 86.38% |
| DTAPHEPBIP | 365 | 0.12% | 86.50% |
| FLU4+VARZOS | 358 | 0.12% | 86.63% |
| DTAP+IPV | 353 | 0.12% | 86.75% |
| FLU3+PPV+TDAP | 337 | 0.11% | 86.86% |
| IPV | 319 | 0.11% | 86.97% |
| DTAP+IPV+MMRV | 304 | 0.10% | 87.07% |
| HPV9+MNQ | 295 | 0.10% | 87.17% |
| FLUR4 | 295 | 0.10% | 87.27% |
| PNC | 288 | 0.10% | 87.37% |
| MEN | 285 | 0.10% | 87.47% |
| DTAP+HIBV | 284 | 0.10% | 87.57% |
| TDAP+VARCEL | 280 | 0.10% | 87.66% |
| DTAP+HEPA | 276 | 0.09% | 87.76% |
| PNC13+VARZOS | 268 | 0.09% | 87.85% |
| HEPA+VARCEL | 266 | 0.09% | 87.94% |
| DTAPIPVHIB+PNC13 | 264 | 0.09% | 88.03% |
| FLU4+TDAP | 261 | 0.09% | 88.12% |
| FLU4+PNC13 | 261 | 0.09% | 88.21% |
| FLUA3+PPV | 257 | 0.09% | 88.29% |
| FLU3+HPV4 | 251 | 0.09% | 88.38% |
| HIBV+PNC13 | 247 | 0.08% | 88.46% |
| DTAP+HIBV+PNC13 | 245 | 0.08% | 88.55% |
| MMR+PNC13+VARCEL | 223 | 0.08% | 88.62% |
| HEP+TDAP | 218 | 0.07% | 88.70% |
| FLUX+VARZOS | 212 | 0.07% | 88.77% |
| HEP+HEPA | 211 | 0.07% | 88.84% |
| MMR+TDAP | 192 | 0.07% | 88.91% |
| RV1 | 191 | 0.07% | 88.97% |
| ANTH+TYP | 188 | 0.06% | 89.04% |
| HEPA+MNQ | 186 | 0.06% | 89.10% |
| ANTH+SMALL+TYP | 183 | 0.06% | 89.16% |
| HPV4+TDAP | 180 | 0.06% | 89.22% |
| PNC13+TDAP | 176 | 0.06% | 89.28% |
| DTP | 174 | 0.06% | 89.34% |
| FLU4+HPV9 | 173 | 0.06% | 89.40% |
| FLUX+PNC13 | 171 | 0.06% | 89.46% |
| HEPA+TDAP | 169 | 0.06% | 89.52% |
| TYP+YF | 167 | 0.06% | 89.57% |
| ANTH+SMALL | 167 | 0.06% | 89.63% |
| FLUR3 | 166 | 0.06% | 89.69% |
| HEPA+HPV9 | 164 | 0.06% | 89.74% |
| HEPA+HPV4+MNQ | 164 | 0.06% | 89.80% |
| MNQ+VARCEL | 163 | 0.06% | 89.85% |
| HEPA+MNQ+TDAP | 162 | 0.06% | 89.91% |
| DTAP+MMR | 161 | 0.05% | 89.96% |
| DTAPIPV+FLU4+MMRV | 159 | 0.05% | 90.02% |
| FLU3+HEPA | 155 | 0.05% | 90.07% |
| DTAP+MMR+VARCEL | 154 | 0.05% | 90.12% |
| HEPA+HPV4+MNQ+TDAP | 153 | 0.05% | 90.18% |
| FLUX(H1N1) | 152 | 0.05% | 90.23% |
| HPV2 | 150 | 0.05% | 90.28% |
| HEPA+MMRV | 150 | 0.05% | 90.33% |
| DTAPIPV+HEPA+MMRV | 149 | 0.05% | 90.38% |
| HPV4+MNQ+TDAP+VARCEL | 148 | 0.05% | 90.43% |
| HEPA+HIBV+MMR+PNC13+VARCEL | 148 | 0.05% | 90.48% |
| DTAPIPVHIB+PNC13+RV1 | 148 | 0.05% | 90.53% |
| HPV9+MENB | 147 | 0.05% | 90.58% |
| DTAP+VARCEL | 147 | 0.05% | 90.63% |
| DTAP+HIBV+IPV+PNC13+RV5 | 147 | 0.05% | 90.68% |
| HPV4+VARCEL | 142 | 0.05% | 90.73% |
| FLU4+HEPA | 142 | 0.05% | 90.78% |
| HEPA+PNC13 | 141 | 0.05% | 90.83% |
| HEP+MMR | 136 | 0.05% | 90.87% |
| HEPA+MMR | 132 | 0.04% | 90.92% |
| DTAPIPVHIB+HEP+PNC13 | 132 | 0.04% | 90.96% |
| FLU3+PPV+VARZOS | 131 | 0.04% | 91.01% |
| DTAPHEPBIP+PNC13 | 130 | 0.04% | 91.05% |
| HIBV+MMR+PNC13+VARCEL | 128 | 0.04% | 91.09% |
| HEPA+TYP | 127 | 0.04% | 91.14% |
| DTAPIPV+MMR | 127 | 0.04% | 91.18% |
| HEPA+MNQ+TDAP+VARCEL | 126 | 0.04% | 91.22% |
| HEP+VARCEL | 126 | 0.04% | 91.27% |
| DTAP+HEPA+HIBV | 123 | 0.04% | 91.31% |
| DTAPIPVHIB+PNC+RV5 | 120 | 0.04% | 91.35% |
| DTAPIPVHIB+HEP+PNC+RV5 | 117 | 0.04% | 91.39% |
| DTAPHEPBIP+HIBV+PNC+RV5 | 116 | 0.04% | 91.43% |
| DTAPIPV+HEPA+MMR+VARCEL | 115 | 0.04% | 91.47% |
| FLUX+TDAP | 113 | 0.04% | 91.51% |
| PPV+TDAP+VARZOS | 111 | 0.04% | 91.54% |
| FLU3+HEPA+MMR+VARCEL | 111 | 0.04% | 91.58% |
| DTAP+HEPA+HIBV+MMR+PNC13+VARCEL | 109 | 0.04% | 91.62% |
| DTAP+IPV+MMR | 108 | 0.04% | 91.66% |
| HIBV+RV5 | 107 | 0.04% | 91.69% |
| HBHEPB | 105 | 0.04% | 91.73% |
| HEP+RV5 | 104 | 0.04% | 91.76% |
| FLU4+PPV+TDAP | 102 | 0.03% | 91.80% |
| FLU3+MNQ | 100 | 0.03% | 91.83% |
| Other | 23980 | 8.17% | 100.00% |

Table 6. The highly frequent AEs in VAERS, 2011-2018

| **Adverse Event** | **No.** | **%** | **Total (%)** |
| --- | --- | --- | --- |
| Injection site erythema | 37161 | 4.29% | 4.29% |
| Pyrexia | 31686 | 3.66% | 7.94% |
| Injection site swelling | 27864 | 3.21% | 11.16% |
| Injection site pain | 27717 | 3.20% | 14.35% |
| Pain | 24062 | 2.78% | 17.13% |
| Erythema | 22230 | 2.56% | 19.69% |
| Pain in extremity | 20178 | 2.33% | 22.02% |
| Headache | 17737 | 2.05% | 24.07% |
| Injection site warmth | 15744 | 1.82% | 25.88% |
| Rash | 15572 | 1.80% | 27.68% |
| Chills | 13071 | 1.51% | 29.19% |
| Nausea | 12864 | 1.48% | 30.67% |
| Dizziness | 12115 | 1.40% | 32.07% |
| Swelling | 11579 | 1.34% | 33.41% |
| Fatigue | 11441 | 1.32% | 34.73% |
| Pruritus | 11231 | 1.30% | 36.02% |
| Urticaria | 10346 | 1.19% | 37.22% |
| Vomiting | 9645 | 1.11% | 38.33% |
| Skin warm | 8929 | 1.03% | 39.36% |
| Herpes zoster | 8744 | 1.01% | 40.37% |
| Injection site pruritus | 7939 | 0.92% | 41.28% |
| Myalgia | 7771 | 0.90% | 42.18% |
| Peripheral swelling | 7131 | 0.82% | 43.00% |
| Asthenia | 6560 | 0.76% | 43.76% |
| Malaise | 6403 | 0.74% | 44.50% |
| Arthralgia | 6276 | 0.72% | 45.22% |
| Dyspnoea | 6236 | 0.72% | 45.94% |
| Injection site rash | 5640 | 0.65% | 46.59% |
| Injection site induration | 5559 | 0.64% | 47.23% |
| Diarrhoea | 5349 | 0.62% | 47.85% |
| Injection site reaction | 5271 | 0.61% | 48.46% |
| Rash erythematous | 5253 | 0.61% | 49.06% |
| Paraesthesia | 5115 | 0.59% | 49.65% |
| Syncope | 5004 | 0.58% | 50.23% |
| Hypoaesthesia | 4877 | 0.56% | 50.79% |
| Cough | 4715 | 0.54% | 51.34% |
| Mobility decreased | 4656 | 0.54% | 51.87% |
| Injected limb mobility decreased | 4111 | 0.47% | 52.35% |
| Immediate post-injection reaction | 4088 | 0.47% | 52.82% |
| Musculoskeletal pain | 3920 | 0.45% | 53.27% |
| Cellulitis | 3863 | 0.45% | 53.72% |
| Rash vesicular | 3851 | 0.44% | 54.16% |
| Tremor | 3644 | 0.42% | 54.58% |
| Hyperhidrosis | 3634 | 0.42% | 55.00% |
| Tenderness | 3580 | 0.41% | 55.42% |
| Influenza like illness | 3495 | 0.40% | 55.82% |
| Loss of consciousness | 3419 | 0.39% | 56.21% |
| Oedema peripheral | 3402 | 0.39% | 56.61% |
| Rash generalised | 3373 | 0.39% | 56.99% |
| Pallor | 3258 | 0.38% | 57.37% |
| Neck pain | 3150 | 0.36% | 57.73% |
| Seizure | 3143 | 0.36% | 58.10% |
| Blister | 3143 | 0.36% | 58.46% |
| Decreased appetite | 3053 | 0.35% | 58.81% |
| Muscular weakness | 3040 | 0.35% | 59.16% |
| Musculoskeletal stiffness | 2926 | 0.34% | 59.50% |
| Feeling abnormal | 2912 | 0.34% | 59.84% |
| Body temperature increased | 2865 | 0.33% | 60.17% |
| Irritability | 2847 | 0.33% | 60.49% |
| Oropharyngeal pain | 2823 | 0.33% | 60.82% |
| Rash pruritic | 2766 | 0.32% | 61.14% |
| Crying | 2766 | 0.32% | 61.46% |
| Local reaction | 2724 | 0.31% | 61.77% |
| Back pain | 2723 | 0.31% | 62.09% |
| Insomnia | 2574 | 0.30% | 62.38% |
| Feeling hot | 2549 | 0.29% | 62.68% |
| Induration | 2480 | 0.29% | 62.96% |
| Lethargy | 2431 | 0.28% | 63.24% |
| Exposure during pregnancy | 2418 | 0.28% | 63.52% |
| Loss of personal independence in daily activities | 2352 | 0.27% | 63.79% |
| Blood test | 2348 | 0.27% | 64.06% |
| Hypersensitivity | 2313 | 0.27% | 64.33% |
| Lymphadenopathy | 2245 | 0.26% | 64.59% |
| Injection site mass | 2175 | 0.25% | 64.84% |
| Fall | 2086 | 0.24% | 65.08% |
| Chest pain | 2056 | 0.24% | 65.32% |
| Swelling face | 2016 | 0.23% | 65.55% |
| Burning sensation | 1990 | 0.23% | 65.78% |
| Gait disturbance | 1914 | 0.22% | 66.00% |
| Injection site urticaria | 1885 | 0.22% | 66.22% |
| Abdominal pain upper | 1869 | 0.22% | 66.44% |
| Rash macular | 1823 | 0.21% | 66.65% |
| Condition aggravated | 1818 | 0.21% | 66.86% |
| Full blood count | 1787 | 0.21% | 67.06% |
| Chest discomfort | 1727 | 0.20% | 67.26% |
| Discomfort | 1712 | 0.20% | 67.46% |
| Laboratory test | 1702 | 0.20% | 67.65% |
| Vaccination complication | 1696 | 0.20% | 67.85% |
| Rhinorrhoea | 1668 | 0.19% | 68.04% |
| Injection site cellulitis | 1644 | 0.19% | 68.23% |
| Contusion | 1637 | 0.19% | 68.42% |
| Unevaluable event | 1603 | 0.18% | 68.61% |
| Nuclear magnetic resonance imaging | 1584 | 0.18% | 68.79% |
| Heart rate increased | 1568 | 0.18% | 68.97% |
| Somnolence | 1566 | 0.18% | 69.15% |
| Abdominal pain | 1534 | 0.18% | 69.33% |
| Injection site nodule | 1508 | 0.17% | 69.50% |
| Injection site bruising | 1498 | 0.17% | 69.67% |
| Muscle spasms | 1434 | 0.17% | 69.84% |
| Full blood count normal | 1432 | 0.17% | 70.00% |
| Wheezing | 1423 | 0.16% | 70.17% |
| Inflammation | 1395 | 0.16% | 70.33% |
| Flushing | 1378 | 0.16% | 70.49% |
| Joint range of motion decreased | 1359 | 0.16% | 70.65% |
| Lip swelling | 1345 | 0.16% | 70.80% |
| Injection site inflammation | 1338 | 0.15% | 70.96% |
| Unresponsive to stimuli | 1312 | 0.15% | 71.11% |
| Throat tightness | 1292 | 0.15% | 71.26% |
| Influenza | 1291 | 0.15% | 71.40% |
| Injection site vesicles | 1280 | 0.15% | 71.55% |
| Skin lesion | 1260 | 0.15% | 71.70% |
| Guillain-Barre syndrome | 1247 | 0.14% | 71.84% |
| Vision blurred | 1229 | 0.14% | 71.98% |
| Laboratory test normal | 1204 | 0.14% | 72.12% |
| Eye swelling | 1173 | 0.14% | 72.26% |
| Abdominal discomfort | 1169 | 0.13% | 72.39% |
| Sleep disorder | 1160 | 0.13% | 72.53% |
| Feeling cold | 1152 | 0.13% | 72.66% |
| Pruritus generalised | 1141 | 0.13% | 72.79% |
| Dyskinesia | 1129 | 0.13% | 72.92% |
| Throat irritation | 1116 | 0.13% | 73.05% |
| Palpitations | 1111 | 0.13% | 73.18% |
| Pharyngeal oedema | 1089 | 0.13% | 73.30% |
| White blood cell count increased | 1062 | 0.12% | 73.43% |
| Underdose | 1054 | 0.12% | 73.55% |
| Ocular hyperaemia | 1037 | 0.12% | 73.67% |
| X-ray | 1006 | 0.12% | 73.78% |
| Dysphagia | 1008 | 0.12% | 73.90% |
| Chest X-ray normal | 1002 | 0.12% | 74.02% |
| Other | 641594 | 25.98% | 100.00% |

Table 7. VAERS SAE report categories, 2011-2018^[[2]](#footnote-3)^

|  | **2011** | | **2012** | | **2013** | | **2014** | | **2015** | |
| --- | --- | --- | --- | --- | --- | --- | --- | --- | --- | --- |
|  | **No.** | **(%)** | **No.** | **(%)** | **No.** | **(%)** | **No.** | **(%)** | **No.** | **(%)** |
| **Death** | 172 | 8.52 | 164 | 8.59 | 127 | 7.02 | 131 | 6.97 | 148 | 7.64 |
| **Disability** | 345 | 17.10 | 344 | 18.01 | 341 | 18.85 | 453 | 24.11 | 435 | 22.46 |
| **Hospitalization** | 1510 | 74.83 | 1390 | 72.77 | 1312 | 72.53 | 1320 | 70.25 | 1332 | 68.77 |
| **Life Threatening** | 431 | 21.36 | 399 | 20.89 | 422 | 23.33 | 424 | 22.57 | 425 | 21.94 |
| **Prolonged Hospitalization** | 151 | 7.48 | 192 | 10.05 | 184 | 10.17 | 139 | 7.40 | 94 | 4.85 |
| **Total SAEs** | **2018** | **100** | **1910** | **100** | **1809** | **100** | **1879** | **100** | **1937** | **100** |

|  | **2016** | | **2017** | | **2018** | | **Total** | |
| --- | --- | --- | --- | --- | --- | --- | --- | --- |
|  | **No.** | **(%)** | **No.** | **(%)** | **No.** | **(%)** | **No.** | **(%)** |
| **Death** | 176 | 9.03 | 120 | 6.54 | 166 | 5.94 | 1204 | 7.46 |
| **Disability** | 466 | 23.90 | 573 | 31.24 | 861 | 30.83 | 3818 | 23.67 |
| **Hospitalization** | 1306 | 66.97 | 1193 | 65.05 | 2002 | 71.68 | 11365 | 70.46 |
| **Life Threatening** | 437 | 22.41 | 354 | 19.30 | 388 | 13.89 | 3280 | 20.33 |
| **Prolonged Hospitalization** | 29 | 1.49 | 21 | 1.15 | 13 | 0.47 | 823 | 5.10 |
| **Total SAEs** | **1950** | **100** | **1834** | **100** | **2793** | **100** | **16130** | **100** |

Table 8. VAERS SAE reports by vaccine combinations, 2011-2018^[[3]](#footnote-4)^

| **Vaccine Combination** | **No.** | **(%)** | **Total (%)** |
| --- | --- | --- | --- |
| FLU3 | 2423 | 15.02% | 15.02% |
| VARZOS | 1732 | 10.74% | 25.76% |
| PPV | 901 | 5.59% | 31.35% |
| HPV4 | 826 | 5.12% | 36.47% |
| FLU4 | 786 | 4.87% | 41.34% |
| FLUX | 518 | 3.21% | 44.55% |
| PNC13 | 507 | 3.14% | 47.69% |
| TDAP | 481 | 2.98% | 50.68% |
| FLU3+PPV | 313 | 1.94% | 52.62% |
| HEP | 304 | 1.88% | 54.50% |
| DTAPHEPBIP+HIBV+PNC13+RV5 | 289 | 1.79% | 56.29% |
| MMR | 259 | 1.61% | 57.90% |
| DTAPIPVHIB+PNC13+RV5 | 247 | 1.53% | 59.43% |
| DTAPHEPBIP+HIBV+PNC13+RV1 | 243 | 1.51% | 60.94% |
| HPV9 | 201 | 1.25% | 62.18% |
| DTAPIPVHIB+HEP+PNC13+RV5 | 181 | 1.12% | 63.30% |
| VARCEL | 168 | 1.04% | 64.35% |
| HEPA | 130 | 0.81% | 65.15% |
| RV5 | 125 | 0.77% | 65.93% |
| MNQ | 124 | 0.77% | 66.70% |
| ANTH | 118 | 0.73% | 67.43% |
| FLUN3 | 117 | 0.73% | 68.15% |
| DTAP | 117 | 0.73% | 68.88% |
| FLUN4 | 98 | 0.61% | 69.49% |
| SMALL | 95 | 0.59% | 70.07% |
| FLU4+PPV | 77 | 0.48% | 70.55% |
| FLUX+PPV | 73 | 0.45% | 71.00% |
| MMR+VARCEL | 72 | 0.45% | 71.45% |
| MENB | 72 | 0.45% | 71.90% |
| HEPA+MMR+VARCEL | 68 | 0.42% | 72.32% |
| DTAPHEPBIP+HIBV+PNC13 | 68 | 0.42% | 72.74% |
| FLUC4 | 66 | 0.41% | 73.15% |
| FLU3+PNC13 | 66 | 0.41% | 73.56% |
| RAB | 59 | 0.37% | 73.92% |
| FLU3+TDAP | 58 | 0.36% | 74.28% |
| HPVX | 57 | 0.35% | 74.64% |
| MMRV | 54 | 0.33% | 74.97% |
| DTAPIPV+MMRV | 53 | 0.33% | 75.30% |
| PPV+TDAP | 50 | 0.31% | 75.61% |
| HEPA+MMR+PNC13+VARCEL | 48 | 0.30% | 75.91% |
| FLU3+VARZOS | 46 | 0.29% | 76.19% |
| MNQ+TDAP | 45 | 0.28% | 76.47% |
| DTAPIPVHIB+HEP+PNC13+RV1 | 45 | 0.28% | 76.75% |
| DTAP+HIBV+IPV+PNC13+RV5 | 45 | 0.28% | 77.03% |
| UNK | 41 | 0.25% | 77.28% |
| FLU(H1N1) | 41 | 0.25% | 77.54% |
| DTAPIPVHIB+PNC13+RV1 | 41 | 0.25% | 77.79% |
| DTAPIPVHIB | 40 | 0.25% | 78.04% |
| DTAPIPV+MMR+VARCEL | 40 | 0.25% | 78.29% |
| TD | 39 | 0.24% | 78.53% |
| YF | 37 | 0.23% | 78.76% |
| MEN | 36 | 0.22% | 78.98% |
| HPV4+MNQ+TDAP | 36 | 0.22% | 79.21% |
| FLUX(H1N1) | 36 | 0.22% | 79.43% |
| DTAPIPVHIB+PNC13 | 36 | 0.22% | 79.65% |
| DTAPHEPBIP+HIBV+PNC+RV5 | 35 | 0.22% | 79.87% |
| ANTH+SMALL | 35 | 0.22% | 80.09% |
| HIBV+PNC13 | 34 | 0.21% | 80.30% |
| HIBV | 34 | 0.21% | 80.51% |
| DTAP+IPV+MMR+VARCEL | 34 | 0.21% | 80.72% |
| PNC | 33 | 0.20% | 80.92% |
| HPV4+MNQ | 33 | 0.20% | 81.13% |
| HEPAB | 32 | 0.20% | 81.33% |
| FLU3+PPV+TDAP | 29 | 0.18% | 81.51% |
| FLU3+HPV4 | 29 | 0.18% | 81.69% |
| DTAP+HIBV+PNC13 | 29 | 0.18% | 81.87% |
| FLUC3 | 28 | 0.17% | 82.04% |
| FLUA3 | 28 | 0.17% | 82.21% |
| DTP | 28 | 0.17% | 82.39% |
| ANTH+SMALL+TYP | 28 | 0.17% | 82.56% |
| HEPA+HPV4 | 27 | 0.17% | 82.73% |
| DTAPHEPBIP+PNC13+RV5 | 27 | 0.17% | 82.90% |
| FLU4+TDAP | 26 | 0.16% | 83.06% |
| TTOX | 25 | 0.15% | 83.21% |
| HPV9+MNQ+TDAP | 25 | 0.15% | 83.37% |
| DTAPIPVHIB+PNC+RV5 | 25 | 0.15% | 83.52% |
| DTAPHEPBIP+PNC13 | 24 | 0.15% | 83.67% |
| DTAPHEPBIP | 24 | 0.15% | 83.82% |
| RV1 | 23 | 0.14% | 83.96% |
| DTAPIPVHIB+HEP+PNC+RV5 | 23 | 0.14% | 84.10% |
| TYP | 22 | 0.14% | 84.24% |
| PPV+VARZOS | 22 | 0.14% | 84.38% |
| DTAP+HIBV | 22 | 0.14% | 84.51% |
| MMR+PNC13+VARCEL | 21 | 0.13% | 84.64% |
| FLU3+HEPA | 21 | 0.13% | 84.77% |
| DTAP+IPV | 20 | 0.12% | 84.90% |
| DTAPIPVHIB+HEP+PNC13 | 19 | 0.12% | 85.02% |
| DTAPIPV | 19 | 0.12% | 85.13% |
| DTAP+HEPA | 19 | 0.12% | 85.25% |
| FLU4+PNC13 | 18 | 0.11% | 85.36% |
| DTAP+HEP+HIBV+IPV+PNC13+RV5 | 18 | 0.11% | 85.47% |
| MENB+MNQ | 17 | 0.11% | 85.58% |
| DTAPHEPBIP+HIBV+PNC+RV1 | 17 | 0.11% | 85.69% |
| ANTH+TYP | 17 | 0.11% | 85.79% |
| TDAP+VARZOS | 16 | 0.10% | 85.89% |
| MNQ+TDAP+VARCEL | 16 | 0.10% | 85.99% |
| FLU3+HEPA+MMR+VARCEL | 16 | 0.10% | 86.09% |
| HIBV+MMR+VARCEL | 15 | 0.09% | 86.18% |
| DTAPHEPBIP+FLU3+HIBV+PNC13 | 15 | 0.09% | 86.27% |
| HEPA+HPV9 | 14 | 0.09% | 86.36% |
| DTAP+HEPA+HIBV+MMR+PNC13+VARCEL | 14 | 0.09% | 86.45% |
| HIBV+MMR | 13 | 0.08% | 86.53% |
| HEPA+TDAP | 13 | 0.08% | 86.61% |
| HEPA+HPV4+MNQ+TDAP | 13 | 0.08% | 86.69% |
| FLU4+VARZOS | 13 | 0.08% | 86.77% |
| FLU4+HPV9 | 13 | 0.08% | 86.85% |
| HEPA+MMR | 12 | 0.07% | 86.92% |
| FLU4+HEPA | 12 | 0.07% | 87.00% |
| DTAPIPVHIB+FLU3+PNC13+RV5 | 12 | 0.07% | 87.07% |
| DTAP+HIBV+MMR | 12 | 0.07% | 87.15% |
| HPV4+TDAP | 11 | 0.07% | 87.22% |
| HEPA+PNC13 | 11 | 0.07% | 87.28% |
| HEPA+MMRV | 11 | 0.07% | 87.35% |
| HEPA+HPV4+MNQ | 11 | 0.07% | 87.42% |
| HEP+HEPA | 11 | 0.07% | 87.49% |
| FLU4+PPV+TDAP | 11 | 0.07% | 87.56% |
| DTAP+IPV+MMRV | 11 | 0.07% | 87.63% |
| DTAP+HEPA+HIBV | 11 | 0.07% | 87.69% |
| DTAP+FLU3+HIBV+PNC13 | 11 | 0.07% | 87.76% |
| IPV | 10 | 0.06% | 87.82% |
| HPV9+MNQ | 10 | 0.06% | 87.89% |
| HIBV+MMR+PNC13+VARCEL | 10 | 0.06% | 87.95% |
| HEPA+HIBV+MMR+PNC13+VARCEL | 10 | 0.06% | 88.01% |
| FLUX+PNC13 | 10 | 0.06% | 88.07% |
| FLU3+HEP | 10 | 0.06% | 88.13% |
| DTAPHEPBIP+HIBV+PNC | 10 | 0.06% | 88.20% |
| DTAP+RV5 | 10 | 0.06% | 88.26% |
| DTAP+MMR+VARCEL | 10 | 0.06% | 88.32% |
| DTAP+MMR | 10 | 0.06% | 88.38% |
| Other | 1874 | 11.62% | 100.00% |

Table 9. The highly frequent AEs for SAEs in VAERS, 2011-2018

| **Adverse Event** | **No.** | **%** | **Total (%)** |
| --- | --- | --- | --- |
| Pyrexia | 2786 | 2.44% | 2.44% |
| Pain | 1403 | 1.23% | 3.66% |
| Vomiting | 1336 | 1.17% | 4.83% |
| Headache | 1272 | 1.11% | 5.94% |
| Dyspnoea | 1202 | 1.05% | 6.99% |
| Pain in extremity | 1082 | 0.95% | 7.94% |
| Asthenia | 1053 | 0.92% | 8.86% |
| Guillain-Barre syndrome | 989 | 0.86% | 9.73% |
| Hypoaesthesia | 978 | 0.86% | 10.58% |
| Seizure | 976 | 0.85% | 11.43% |
| Death | 972 | 0.85% | 12.28% |
| Herpes zoster | 943 | 0.82% | 13.11% |
| Fatigue | 932 | 0.81% | 13.92% |
| Blood test | 890 | 0.78% | 14.70% |
| Muscular weakness | 875 | 0.77% | 15.47% |
| Nausea | 848 | 0.74% | 16.21% |
| Nuclear magnetic resonance imaging | 822 | 0.72% | 16.93% |
| Dizziness | 803 | 0.70% | 17.63% |
| Laboratory test | 778 | 0.68% | 18.31% |
| Injection site pain | 772 | 0.68% | 18.98% |
| Rash | 765 | 0.67% | 19.65% |
| Paraesthesia | 748 | 0.65% | 20.31% |
| Malaise | 685 | 0.60% | 20.91% |
| Chills | 656 | 0.57% | 21.48% |
| Immunoglobulin therapy | 604 | 0.53% | 22.01% |
| Lumbar puncture | 603 | 0.53% | 22.54% |
| Injection site erythema | 595 | 0.52% | 23.06% |
| Erythema | 589 | 0.52% | 23.57% |
| Intensive care | 565 | 0.49% | 24.07% |
| Diarrhoea | 558 | 0.49% | 24.55% |
| White blood cell count increased | 552 | 0.48% | 25.04% |
| Computerised tomogram | 546 | 0.48% | 25.51% |
| Arthralgia | 546 | 0.48% | 25.99% |
| Gait disturbance | 537 | 0.47% | 26.46% |
| Injection site swelling | 528 | 0.46% | 26.92% |
| Cough | 499 | 0.44% | 27.36% |
| Myalgia | 490 | 0.43% | 27.79% |
| Loss of personal independence in daily activities | 480 | 0.42% | 28.21% |
| Intussusception | 465 | 0.41% | 28.61% |
| Laboratory test normal | 454 | 0.40% | 29.01% |
| Chest pain | 449 | 0.39% | 29.40% |
| Mobility decreased | 444 | 0.39% | 29.79% |
| Full blood count | 435 | 0.38% | 30.17% |
| Gait inability | 418 | 0.37% | 30.54% |
| Back pain | 409 | 0.36% | 30.89% |
| Tremor | 408 | 0.36% | 31.25% |
| Pneumonia | 402 | 0.35% | 31.60% |
| Swelling | 398 | 0.35% | 31.95% |
| Cellulitis | 386 | 0.34% | 32.29% |
| Lethargy | 381 | 0.33% | 32.62% |
| Unresponsive to stimuli | 380 | 0.33% | 32.95% |
| Urticaria | 377 | 0.33% | 33.28% |
| Condition aggravated | 375 | 0.33% | 33.61% |
| X-ray | 363 | 0.32% | 33.93% |
| Full blood count normal | 354 | 0.31% | 34.24% |
| Crying | 354 | 0.31% | 34.55% |
| Blood culture negative | 343 | 0.30% | 34.85% |
| Fall | 339 | 0.30% | 35.14% |
| Electroencephalogram | 334 | 0.29% | 35.44% |
| Decreased appetite | 331 | 0.29% | 35.73% |
| Irritability | 330 | 0.29% | 36.01% |
| Nuclear magnetic resonance imaging normal | 324 | 0.28% | 36.30% |
| Syncope | 321 | 0.28% | 36.58% |
| Computerised tomogram normal | 321 | 0.28% | 36.86% |
| Loss of consciousness | 313 | 0.27% | 37.13% |
| Injected limb mobility decreased | 290 | 0.25% | 37.39% |
| Pruritus | 289 | 0.25% | 37.64% |
| Abdominal pain | 289 | 0.25% | 37.89% |
| Immediate post-injection reaction | 286 | 0.25% | 38.14% |
| Platelet count decreased | 284 | 0.25% | 38.39% |
| Chest X-ray normal | 284 | 0.25% | 38.64% |
| Feeling abnormal | 283 | 0.25% | 38.89% |
| Electrocardiogram | 282 | 0.25% | 39.13% |
| Electroencephalogram normal | 279 | 0.24% | 39.38% |
| Musculoskeletal pain | 276 | 0.24% | 39.62% |
| Peripheral swelling | 273 | 0.24% | 39.86% |
| Chest X-ray | 265 | 0.23% | 40.09% |
| Musculoskeletal stiffness | 261 | 0.23% | 40.32% |
| Endotracheal intubation | 257 | 0.22% | 40.54% |
| Nuclear magnetic resonance imaging brain normal | 251 | 0.22% | 40.76% |
| Computerised tomogram head | 250 | 0.22% | 40.98% |
| Body temperature increased | 249 | 0.22% | 41.20% |
| Metabolic function test | 247 | 0.22% | 41.41% |
| Dehydration | 247 | 0.22% | 41.63% |
| Neck pain | 246 | 0.22% | 41.84% |
| Insomnia | 245 | 0.21% | 42.06% |
| Nuclear magnetic resonance imaging abnormal | 242 | 0.21% | 42.27% |
| Blood test normal | 242 | 0.21% | 42.48% |
| Influenza like illness | 240 | 0.21% | 42.69% |
| Pallor | 239 | 0.21% | 42.90% |
| Paralysis | 237 | 0.21% | 43.11% |
| CSF protein increased | 237 | 0.21% | 43.31% |
| Autism spectrum disorder | 237 | 0.21% | 43.52% |
| Vaccination complication | 233 | 0.20% | 43.73% |
| Hyperhidrosis | 228 | 0.20% | 43.92% |
| Balance disorder | 227 | 0.20% | 44.12% |
| Vaccination failure | 226 | 0.20% | 44.32% |
| Urine analysis normal | 224 | 0.20% | 44.52% |
| C-reactive protein increased | 224 | 0.20% | 44.71% |
| Urine analysis | 223 | 0.19% | 44.91% |
| Other | 63006 | 55.09% | 100.00% |

1. For the full name of vaccines please check VAERS DATA USE GUIDE at <https://vaers.hhs.gov/docs/VAERSDataUseGuide_October2017.pdf> [↑](#footnote-ref-2)
2. The total sum of SAE categories in each years is more than the total SAE reports in each year. It is because SAE categories are not mutually exclusive and an SAE could have more than one reason of seriousness. [↑](#footnote-ref-3)
3. For the full name of vaccines please check VAERS DATA USE GUIDE at <https://vaers.hhs.gov/docs/VAERSDataUseGuide_October2017.pdf> [↑](#footnote-ref-4)
