# Supplementary material for: Vaccine hesitancy: evidence from an adverse events following immunization database, and the role of cognitive biases
Source: BMC Public Health. 2021 Sep 16;21:1686. doi: 10.1186/s12889-021-11745-1 (PMC8444164; doi:10.1186/s12889-021-11745-1)
Supplement: Supplementary file 2 — Additional file 2: Supplementary Material 2. Canada Vigilance database results. [file 12889_2021_11745_MOESM2_ESM.docx]

# Supplementary Material 2: Canada Vigilance database results

We applied the same analysis on Canada Vigilance (CV) (1), another publicly available AEFI data source at the country level. Canada has two vaccine surveillance systems: Canadian Adverse Events Following Immunization Surveillance System (CAEFISS) (2) that its data is not available to the public, and CV that its data is publically available. Since 1987, CAEFISS has had the primary responsibility for vaccine surveillance in Canada. However, as of January 2011, it started sharing its responsibility with CV because of a change in regulations that required Market Authorization Holders (MAHs) to report AEFIs to CV (3, 4). Like the paper’s primary study analysis on VAERS, the data covers January 2011 to December 2018 and is publicly available. We also used the reports published by CV (CV reports list) when a more in-depth investigation was needed. The data of CAEFISS is not accessible to the public, so all the analyses on the CAEFISS data are based on the information extracted from CAEFISS reports (‎CAEFISS reports list).

# Methods

CV receives AE reports for drugs and vaccines. In the first step, we identified vaccines in the database. CV does not provide an identification key for vaccines, so we used a combination of different ways to identify all vaccines. First, we extracted all products with “vaccine” in their names or their active ingredient names to make the initial table of vaccines. Next, we created a list of all the product names from the Canada Drug Product Database (5) that their Anatomical Therapeutic Chemical (ATC) code was J07 (ATC code for vaccines). We searched the CV database against this list to add new vaccines to the table. Next, we extracted all vaccines from VAERS and added the new common vaccines between CV and VAERS to the table. Finally, we looked for the ATC code of the products in the vaccine table to validated them. This process resulted in a table of 376 vaccine names and 450 vaccine IDs (Figure 1). limiting the time range of the initial received date of the reports to January 1, 2011, through December 31, 2018 (the period of this study) resulted in 5535 reports. We removed all known duplicate reports (reports concerning the same patient but received from different sources), which resulted in 5384 reports. In each duplicate case, we only kept the last occurrence of the report and removed the others.

Each health product in CV reports has a role which could be “Concomitant,” “Drug Used to Treat Adverse Effect,” or “Suspect.” An AEFI report is an AE report that has at least one vaccine as a suspect. In the next step, we checked all the suspected products in the reports. We found 4397 reports with only vaccines as suspects, 345 reports with at least one vaccine and one non-vaccine product as suspects, and 642 reports that only non-vaccines were the suspects (all vaccines in these reports were concomitant). Since the first and the second group had at least one vaccine as a suspected cause, based on the AEFI definition, we considered them as AEFI reports. Therefore, the total number of AEFI reports submitted to CV with the initial received date from January 1, 2011, through December 31, 2018, is 4742 reports (Figure 1).


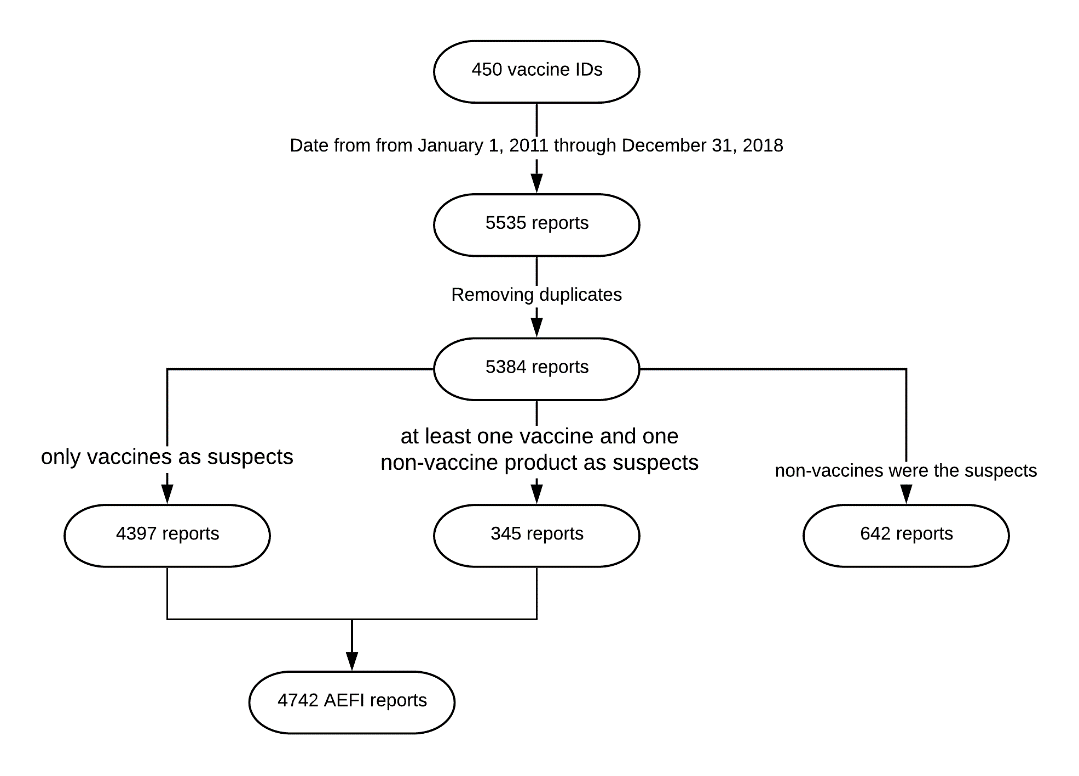


Figure 1. The process of preparing the data from the CV database.

AEs recorded in CV and VAERS are coded in the International Medical Dictionary for Regulatory Activities (MedDRA) Preferred Terms (PTs). PTs in CV are in MedDRA version 22.0; in VAERS, they are from versions 13.1 to 22.0. To provide a consistent view and accurate result, we converted all PTs from all other versions to PTs version 22.0.

# CV Results

**Number of reports.** From January 1, 2011, to December 31, 2018, 32099 reports were collected in Canada by CAEFISS and CV. CV received 4742 (14.77%) and CAEFISS received 27357 (85.23%) reports (6, 7). In the eight years, Canada’s average population-based reporting rate was 112.63 reports per 1 million population. Canada’s average population-based reporting rate was calculated based on the CAEFISS reports (CAEFISS reports list) and the CV results. Canada’s population-based reporting rate in comparison to the U.S. population-based reporting rate from 2011 to 2018 is depicted in Figure 2. The number of serious AEFI reports was not provided in these reports for each year. So we could not provide the trend line for the Serious Adverse Event (SAE) reports.

Figure 2. Population-based AEFI reporting rate in the U.S. and Canada

**Age distribution (Tables and figures, Table 2).** The percentage of children aged <7 years in VAERS and CV are close (VAERS: 15.7%, CV: 16.7%, Table 1), which is the primary age group in the recommended vaccination schedules (8). A notable difference between the age distribution in VAERS and CV is in the ≥65 group (VAERS: 17.56%; CV: 2.64%). In both databases, the 18-64 age group has the highest percentage of reports (VAERS: 31.23%; CV: 38.17%).

Table 1. VAERS and CV reports by age groups, 2011-2018

|  | **VAERS** | | **CV** | |
| --- | --- | --- | --- | --- |
| **System** | **No.** | **(%)** | **No.** | **(%)** |
| **<1** | **14634** | **4.98** | **228** | **4.81** |
| **1-6** | **31497** | **10.73** | **564** | **11.89** |
| **7-17** | **31132** | **10.60** | **336** | **7.09** |
| **18-64** | **91707** | **31.23** | **1810** | **38.17** |
| **≥65** | **51545** | **17.56** | **125** | **2.64** |
| **Unknow** | **73094** | **24.90** | **1679** | **35.41** |

**Sex distribution (‎Tables and figures, Table 3).** More than 50% of the patients are females (VAERS: 51.8%; CV: 56.6%), while the percentage of males is 27.2% in VAERS and 29.8% in CV. The sex of the rest of the reports is either unknown or not specified.

**Age-sex distribution (‎‎Tables and figures, Table 4).** For children aged <7 years and adolescents (7-17 years), the percentage of males (VAERS: 11.71%, CV: 9.42%) and females (VAERS: 11.9%, CV: 8.62%) are close. However, the percentage of adult (18-64) females (VAERS: 22.5%, CV: 27.31%) is higher than adult males (VAERS: 8.03%, CV: 9.83%) and at the peak in both databases.

**Vaccine types.** CV reports involve 58 different vaccine types from 243 different vaccine brands. There are mentions of 1 to 5 different vaccine types in each report. 93.8% of reports involve one, 4.6% involve two, and 1.1% involve three to five vaccine types. The 58 vaccine types entail 294 vaccine type combinations. Thirty-eight highly frequent vaccines or vaccine combinations ( ≥10 reports) comprise 90.3% of all CV reports (‎Tables and figures, Table 5). The top five highly frequent vaccine types are Varicella-Zoster Vaccine (VARZOS: 23%), Influenza Virus Vaccine, Trivalent (FLU3: 11%), Influenza Virus Vaccine (No Brand Name) (FLUX: 6.5%), Pneumococcal Vaccine, Polyvalent (PPV: 6.1%), and Meningococcal Group B Vaccine, rDNA Absorbed (MENB: 5.4%).

The reason for the high number of VARZOS vaccine type (23%) in CV is the same VAERS. In CV, the average number of VARZOS reports per year from 2011 to 2017 was 87, whereas it increased to 477 in 2018. In 2017, a new vaccine brand (Shingrix) in the VARZOS group was released in the U.S. and Canada. In 2018, 88.5% of the VARZOS AEFIs in CV belonged to Shingrix.

**Adverse Events.** In CV, 4742 reports involve 18682 AEs, 80 of which are “No adverse event,” and 612 of them belong to “Medication errors and other product use errors and issues.” After excluding these two groups, 4396 reports involve 17996 AEs from 1822 different AEs. The range of AEs per report is from 1 to 83. Removing the outliers (AEs per report: >11), on average, each report in CV contains 3 AEs and the median is 2 (24.34%). The top 10 highly frequent AEs in CV are Pyrexia (2.51%), Headache (1.97%), Pain in extremity (1.88%), Fatigue (1.78%), Herpes zoster (1.57%), Nausea (1.56%), Pain (1.49%), Erythema (1.39%), Vaccination site erythema (1.33%), and Malaise (1.27%). The highly frequent AEs in CV (≥100) are depicted in the ‎‎Tables and figures section, Table 6.

**Serious Adverse Events (SAE) reports.** Out of 4742 CV reports, 2687 (56.66%) of them were SAE. Of the six reasons for SAE reports in CV, “Other medically important condition” (77.4%) was the main reason (‎Tables and figures, Table 7). Hospitalization (20.4%) and Disability (8.86%) were next. Also, for 2.74% of SAE reports, the reason was death. The top Five vaccine types in CV SAE reports are Varicella-Zoster Vaccine (VARZOS: 24.1%), Influenza Virus Vaccine, Trivalent (FLU3: 9.42%), Influenza Virus Vaccine (No Brand Name) (FLUX: 8.9%), Pneumococcal Vaccine, Polyvalent (PPV: 6.51%), and Human Papillomavirus 4-Valent (HPV4: 4.1%) (‎Tables and figures, Table 8). The top five AEs in CV SAE reports are Pyrexia (2.2%), Herpes zoster (1.84%), Headache (1.7%), Vaccination failure (1.64%), and Fatigue (1.58%) (‎Tables and figures, Table 9). In CV SAE reports, the outcome of 20% recovered, 20.5% of patients were not recovered at the time of reporting, 8.4% recovering, and 47% were unknown.

The percentage of SAE reports in CV is 56.66%. To explain this high percentage, we must consider that, in Canada, CAEFISS collected 85.23% (n=27357) of the reports in 2011-2018, and the community was the source of more than 95% of them. The high contribution of the community in CAEFISS reports reduces the percentage of SAE reports in CAEFISS to 6.4% (n=1742) (3, 6, 7). In CV, MAHs are the main source of reports as they must report SAEs to CV. In 2011-2018, 50.6% (n=2400) of the reports in CV were from MAHs, which the majority of them (70.2%, n=1685) were SAEs. Considering all reports from CV and CAEFISS, the percentage of SAE reports in Canada in 2011-2018 will be 13.8% (n=4429).

Like VAERS, CV and CAEFISS monitor all the AEFI reports and conduct further investigations for the SAE reports. CAEFISS and CV publish reports (‎CV reports list and CAEFISS reports list) to provide an overview of vaccination safety and detail the result of their investigations on SAE reports. In CAEFISS, most of the SAEs were found to be expected, to have alternative explanations not relevant to vaccination, or to be under more investigation. In CV, for all SAE reports including death since 2015, either the information was not enough to adequately assess the causal association with the vaccine (mostly for the reports that MAHs collected them from social media), or in the majority of cases, patients were with underlying medical conditions and a causality association to vaccines were unlikely. For both databases, no vaccine safety signals were identified.

# References of the supplementary material 2

1. Health Canada. Canada Vigilance Program 2018 [Available from: <https://www.canada.ca/en/health-canada/services/drugs-health-products/medeffect-canada/canada-vigilance-program.html>.

2. Health Canada. Canadian Adverse Events Following Immunization Surveillance System (CAEFISS) 2019 [Available from: <https://www.canada.ca/en/public-health/services/immunization/canadian-adverse-events-following-immunization-surveillance-system-caefiss.html>.

3. Law B, Laflèche J, Ahmadipour N, Anyoti H. Canadian Adverse Events Following Immunization Surveillance System (CAEFISS): Annual report for vaccines administered in 2012. Canada communicable disease report= Releve des maladies transmissibles au Canada. 2014;40(Suppl 3):7-23.

4. Ahmadipour N, Watkins K, Fréchette M, Coulby C, Anyoti H, Johnson K. Vaccine safety surveillance in Canada: Reports to CAEFISS, 2013-2016. Can Commun Dis Rep. 2018;44(9):206-14.

5. Drug Product Database: Access the database [Available from: <https://www.canada.ca/en/health-canada/services/drugs-health-products/drug-products/drug-product-database.html>.

6. Johnson K, Anyoti H, Coulby C. Vaccine safety surveillance in Canada: Reports to CAEFISS, 2017. Canada Communicable Disease Report. 2018;44(12):333-9.

7. CAEFISS. Adverse Events Following Immunization (AEFI) Bi-annual Report from July 1 to December 31, 2018. 2019.

8. Zhou W, Pool V, Iskander JK, English-Bullard R, Ball R, Wise RP, et al. Surveillance for safety after immunization: vaccine adverse event reporting system (VAERS)—United States, 1991–2001. MMWR Surveill Summ. 2003;52(1):1-24.

# CV reports list

1. H. P. I. E. Team, “Health Product InfoWatch – November 2015,” in “Health Product InfoWatch,” 2015. Accessed: 7/25/2019. [Online]. Available: <https://www.canada.ca/content/dam/hc-sc/migration/hc-sc/dhp-mps/alt_formats/pdf/medeff/bulletin/hpiw-ivps_2015-11-eng.pdf>
2. H. P. I. E. Team, “Health Product InfoWatch - February 2016,” in “Health Product InfoWatch,” 2016. Accessed: 7/25/2019. [Online]. Available: <https://www.canada.ca/en/health-canada/services/drugs-health-products/medeffect-canada/health-product-infowatch/health-product-infowatch-february-2016.html>
3. H. P. I. E. Team, “Health Product InfoWatch – November 2016,” in “Health Product InfoWatch,” 2016. Accessed: 7/25/2019. [Online]. Available: <https://www.canada.ca/content/dam/hc-sc/migration/hc-sc/dhp-mps/alt_formats/pdf/medeff/bulletin/hpiw-ivps_2016-11-eng.pdf>
4. H. P. I. E. Team, “Health Product InfoWatch – August 2016,” in “Health Product InfoWatch,” 2016. Accessed: 7/25/2019. [Online]. Available: <https://www.canada.ca/content/dam/hc-sc/migration/hc-sc/dhp-mps/alt_formats/pdf/medeff/bulletin/hpiw-ivps_2016-08-eng.pdf>
5. H. P. I. E. Team, “Health Product InfoWatch – May 2016,” in “Health Product InfoWatch,” 2016. Accessed: 7/25/2019. [Online]. Available: <https://www.canada.ca/content/dam/hc-sc/migration/hc-sc/dhp-mps/alt_formats/pdf/medeff/bulletin/hpiw-ivps_2016-05-eng.pdf>
6. H. P. I. E. Team, “Health Product InfoWatch – February 2016,” in “Health Product InfoWatch,” 2016. Accessed: 7/25/2019. [Online]. Available: <https://www.canada.ca/content/dam/hc-sc/migration/hc-sc/dhp-mps/alt_formats/pdf/medeff/bulletin/hpiw-ivps_2016-02-eng.pdf>
7. H. P. I. E. Team, “Health Product InfoWatch – February 2017,” in “Health Product InfoWatch,” 2017. Accessed: 7/25/2019. [Online]. Available: <https://www.canada.ca/content/dam/hc-sc/migration/hc-sc/dhp-mps/alt_formats/pdf/medeff/bulletin/hpiw-ivps_2017-02-eng.pdf>
8. H. P. I. E. Team, “Health Product InfoWatch – May 2017,” in “Health Product InfoWatch,” 2017. Accessed: 7/25/2019. [Online]. Available: <https://www.canada.ca/content/dam/hc-sc/migration/hc-sc/dhp-mps/alt_formats/pdf/medeff/bulletin/hpiw-ivps_2017-05-eng.pdf>
9. H. P. I. E. Team, “Health Product InfoWatch – August 2017,” in “Health Product InfoWatch,” 2017. Accessed: 7/25/2019. [Online]. Available: <https://www.canada.ca/content/dam/hc-sc/documents/services/drugs-health-products/medeffect-canada/health-product-infowatch/health-product-infowatch-august-2017/hpiw-ivps_2017-08-eng.pdf>
10. H. P. I. E. Team, “Health Product InfoWatch – April 2018,” in “Health Product InfoWatch,” 2018. Accessed: 7/25/2019. [Online]. Available: <https://www.canada.ca/content/dam/hc-sc/documents/services/drugs-health-products/medeffect-canada/health-product-infowatch/april-2018/hpiw-ivps_2018-04-eng.pdf>
11. H. P. I. E. Team, “Health Product InfoWatch – July 2018,” in “Health Product InfoWatch,” 2018. Accessed: 7/25/2019. [Online]. Available: <https://www.canada.ca/content/dam/hc-sc/documents/services/drugs-health-products/medeffect-canada/health-product-infowatch/health-product-infowatch-july-2018/hpiw-ivps_2018-07-30-eng.pdf>
12. H. P. I. E. Team, “Health Product InfoWatch – January 2019,” in “Health Product InfoWatch,” 2019. Accessed: 7/25/2019. [Online]. Available: <https://www.canada.ca/content/dam/hc-sc/documents/services/drugs-health-products/medeffect-canada/health-product-infowatch/health-product-infowatch-january-2019/hpiw-ivps_2019-01-eng.pdf>
13. H. P. I. E. Team, “Health Product InfoWatch – June 2019,” in “Health Product InfoWatch,” 2019. Accessed: 7/25/2019. [Online]. Available: https://www.canada.ca/content/dam/hc-sc/documents/services/drugs-health-products/medeffect-canada/health-product-infowatch/health-product-infowatch-june-2019/hpiw-ivps_2019-06-eng.pdf

# CAEFISS reports list^[[1]](#footnote-2)^

1. “Adverse events temporally associated with immunizing agents: 1987 report,” Canadian Medical Association Journal, vol. 141, no. 9, pp. 933-937, 1989.
2. P. Duclos, R. McCarthy, J. Koch, and A. Carter, “Adverse events temporally associated with immunizing agents-1988 report,” Canada Diseases Weekly Report, vol. 16, no. 32, pp. 157-166, 1990.
3. P. Duclos and M. Hardy, “Adverse events temporally associated with immunizing agents: 1989 report,” Can. Med. Assoc. J., vol. 145, pp. 1269-1275, 1991.
4. P. Duclos, R. Pless, J. Koch, and M. Hardy, “Adverse events temporally associated with immunizing agents: 1990 report,” Canadian Family Physician, vol. 39, p. 1907, 1993.
5. A. Bentsi-Enchill, M. Hardy, J. Koch, and P. Duclos, “Adverse events temporally associated with vaccines--1992 report,” Canada communicable disease report= Releve des maladies transmissibles au Canada, vol. 21, no. 13, pp. 117-128, 1995.
6. P. H. A. o. Canada, “Canadian National Report on Immunization, 2006,” Can Commun Dis Rep, vol. 32, pp. 1-44, 2006. [Online]. Available: <https://www.canada.ca/en/public-health/services/reports-publications/canada-communicable-disease-report-ccdr/monthly-issue/2006-32/supplement-canadian-national-report-on-immunization-2006/canadian-national-report-on-immunization-2006.html>.
7. J. A. Bettinger, S. A. Halperin, W. Vaudry, B. Law, and D. W. Scheifele, “The Canadian Immunization Monitoring Program, ACTive (IMPACT): Active surveillance for vaccine adverse events and vaccine-preventable diseases,” Canada Communicable Disease Report, vol. 40, no. Suppl 3, p. 41, 2014.
8. K. Johnson, H. Anyoti, and C. Coulby, “Vaccine safety surveillance in Canada: Reports to CAEFISS, 2017,” Canada Communicable Disease Report, vol. 44, no. 12, pp. 333-339, 2018.
9. B. o. I. D. Division of Immunization, Laboratory Centre for Disease Control, “Canadian National Report on Immunization, 1996,” in “Surveillance of adverse events temporally associated with vaccine administration,” 1997.
10. CAEFISS, “Adverse Events Following Immunization (AEFI) Quarterly Report for 2014 – Q1,” in “CAEFISS quarterly reports,” 2014. Accessed: 7/25/2019. [Online]. Available: <https://www.canada.ca/en/public-health/services/publications/healthy-living/adverse-events-following-immunization-aefi-quarterly-report-2014-q1.html>
11. CAEFISS, “Adverse Events Following Immunization (AEFI) Quarterly Report for 2014 – Q2,” in “CAEFISS quarterly reports,” 2014. Accessed: 7/25/2019. [Online]. Available: <https://www.canada.ca/en/public-health/services/publications/healthy-living/adverse-events-following-immunization-aefi-quarterly-report-2014-q2.html>
12. CAEFISS, “Adverse Events Following Immunization (AEFI) Quarterly Report for 2015 – Q1,” in “CAEFISS quarterly reports,” 2015. Accessed: 7/25/2019. [Online]. Available: <https://www.canada.ca/en/public-health/services/publications/healthy-living/adverse-events-following-immunization-aefi-quarterly-report-2015-q1.html>
13. CAEFISS, “Adverse Events Following Immunization (AEFI) Quarterly Report for 2015 – Q2,” in “CAEFISS quarterly reports,” 2015. Accessed: 7/25/2019. [Online]. Available: <https://www.canada.ca/en/public-health/services/publications/healthy-living/adverse-events-following-immunization-aefi-quarterly-report-2015-q2.html>
14. CAEFISS, “Adverse Events Following Immunization (AEFI) Quarterly Report for 2014 – Q3,” in “CAEFISS quarterly reports,” 2015. Accessed: 7/25/2019. [Online]. Available: <https://www.canada.ca/en/public-health/services/publications/healthy-living/adverse-events-following-immunization-aefi-quarterly-report-2014-q3.html>
15. CAEFISS, “Adverse Events Following Immunization (AEFI) Quarterly Report for 2014 – Q4,” in “CAEFISS quarterly reports,” 2015. Accessed: 7/25/2019. [Online]. Available: <https://www.canada.ca/en/public-health/services/publications/healthy-living/adverse-events-following-immunization-aefi-quarterly-report-2014-q4.html>
16. CAEFISS, “Adverse Events Following Immunization (AEFI) Quarterly Report for 2015 – Q3,” in “CAEFISS quarterly reports,” 2016. Accessed: 7/25/2019. [Online]. Available: <https://www.canada.ca/en/public-health/services/publications/healthy-living/adverse-events-following-immunization-aefi-quarterly-report-2015-q3.html>
17. CAEFISS, “Adverse Events Following Immunization (AEFI) Quarter 4 Report for 2015 – Q4,” in “CAEFISS quarterly reports,” 2016. Accessed: 7/25/2019. [Online]. Available: <https://www.canada.ca/en/public-health/services/publications/healthy-living/adverse-events-following-immunization-aefi-quarter-4-report-2015-october-1-2015-december-31-2015.html>
18. CAEFISS, “Adverse Events Following Immunization (AEFI) Quarter 1 Report for 2016 (January 1 – March 31),” in “CAEFISS quarterly reports,” 2016. Accessed: 7/25/2019. [Online]. Available: <https://www.canada.ca/en/public-health/services/publications/healthy-living/adverse-events-following-immunization-aefi-quarter-1-report-2016-january-1-march-31.html>
19. CAEFISS, “Adverse Events Following Immunization (AEFI) Quarter 2 Report for 2016 (April 1 – June 30),” in “CAEFISS quarterly reports,” 2016. Accessed: 7/25/2019. [Online]. Available: <https://www.canada.ca/en/public-health/services/publications/healthy-living/adverse-events-following-immunization-quarter-2-report-april-1-june-30-2016.html>
20. CAEFISS, “Adverse Events Following Immunization (AEFI) Quarter 3 Report for 2016 (July 1 – September 30),” in “CAEFISS quarterly reports,” 2017. Accessed: 7/25/2019. [Online]. Available: <https://www.canada.ca/en/public-health/services/publications/healthy-living/adverse-events-following-immunization-quarter-3-report-july-1-september-30-2016.html>
21. CAEFISS, “Adverse Events Following Immunization (AEFI) Quarter 4 Report for 2016 (October 1 – December 31),” in “CAEFISS quarterly reports,” 2017. Accessed: 7/25/2019. [Online]. Available: <https://www.canada.ca/en/public-health/services/publications/healthy-living/adverse-events-following-immunization-quarter-4-report-october-1-december-31-2016.html>
22. CAEFISS, “Adverse Events Following Immunization (AEFI) Bi-annual Report from July 1 to December 31, 2018,” 2019. Accessed: 7/25/2019. [Online]. Available: <https://www.canada.ca/en/public-health/services/publications/vaccines-immunization/adverse-events-following-immunization-bi-annual-report-july-1-december-31-2018.html>
23. CAEFISS, “Adverse Events Following Immunization (AEFI) Bi-annual Report from January 1 to June 30, 2018,” 2019. Accessed: 7/25/2019. [Online]. Available: <https://www.canada.ca/en/public-health/services/publications/pseudonode/adverse-events-following-immunization-bi-annual-report-january-1-june-30-2018.html>
24. B. Law, J. Laflèche, N. Ahmadipour, and H. Anyoti, “Canadian Adverse Events Following Immunization Surveillance System (CAEFISS): Annual report for vaccines administered in 2012,” Canada communicable disease report= Releve des maladies transmissibles au Canada, vol. 40, no. Suppl 3, pp. 7-23, 2014.
25. N. Ahmadipour, K. Watkins, M. Fréchette, C. Coulby, H. Anyoti, and K. Johnson, “Vaccine safety surveillance in Canada: Reports to CAEFISS, 2013-2016,” (in eng), Canada communicable disease report = Releve des maladies transmissibles au Canada, vol. 44, no. 9, pp. 206-214, 2018, doi: 10.14745/ccdr.v44i09a04.

# Tables and figures

Table 1. The annual number of AEFI reports for VAERS, CV, and CAEFISS, 2011-2018

| Year | CAEFISS reports | CV reports | Canada Total report | VAERS reports |
| --- | --- | --- | --- | --- |
| 2011 | 3835 | 460 | 4295 | 25336 |
| 2012 | 4001 | 451 | 4452 | 26590 |
| 2013 | 3417 | 512 | 3929 | 29651 |
| 2014 | 3437 | 702 | 4139 | 34307 |
| 2015 | 3302 | 719 | 4021 | 44335 |
| 2016 | 3180 | 487 | 3667 | 45561 |
| 2017 | 3063 | 483 | 3546 | 38800 |
| 2018 | 3122 | 928 | 4050 | 49029 |
| Total | **27357** | **4742** | **32099** | **293609** |

Table 2. CV AEFI reports by age groups, 2011-2018

|  | **Age** | | | | | | | | | | | | | |
| --- | --- | --- | --- | --- | --- | --- | --- | --- | --- | --- | --- | --- | --- | --- |
|  | **<1** | | **1-6** | | **7-17** | | **18-64** | | **≥65** | | **Unknown** | | **Total** | |
| **Year** | No. | (%) | No. | (%) | No. | (%) | No. | (%) | No. | (%) | No. | (%) | **No.** | **(%)** |
| **2011** | 50 | 10.87 | 111 | 24.13 | 43 | 9.35 | 137 | 29.78 | 10 | 2.17 | 109 | 23.70 | **460** | **100** |
| **2012** | 44 | 9.76 | 75 | 16.63 | 47 | 10.42 | 166 | 36.81 | 10 | 2.22 | 109 | 24.17 | **451** | **100** |
| **2013** | 40 | 7.81 | 45 | 8.79 | 24 | 4.69 | 226 | 44.14 | 9 | 1.76 | 168 | 32.81 | **512** | **100** |
| **2014** | 23 | 3.28 | 107 | 15.24 | 56 | 7.98 | 290 | 41.31 | 13 | 1.85 | 213 | 30.34 | **702** | **100** |
| **2015** | 28 | 3.89 | 71 | 9.87 | 69 | 9.60 | 262 | 36.44 | 16 | 2.23 | 273 | 37.97 | **719** | **100** |
| **2016** | 21 | 4.31 | 51 | 10.47 | 28 | 5.75 | 201 | 41.27 | 17 | 3.49 | 169 | 34.70 | **487** | **100** |
| **2017** | 9 | 1.86 | 50 | 10.35 | 31 | 6.42 | 211 | 43.69 | 19 | 3.93 | 163 | 33.75 | **483** | **100** |
| **2018** | 13 | 1.40 | 54 | 5.82 | 38 | 4.09 | 317 | 34.16 | 31 | 3.34 | 475 | 51.19 | **928** | **100** |
| **Total** | **228** | **4.81** | **564** | **11.89** | **336** | **7.09** | **1810** | **38.17** | **125** | **2.64** | **1679** | **35.41** | **4742** | **100** |

Table 3. CV AEFI reports by sex groups, 2011-2018‎

|  | **SEX** | | | | | | | | | |
| --- | --- | --- | --- | --- | --- | --- | --- | --- | --- | --- |
|  | **Female** | | **Male** | | **Not specified** | | **Unknown** | | **Total** | |
|  | No. | (%) | No. | (%) | No. | (%) | No. | (%) | **No.** | **(%)** |
| **2011** | 227 | 49.35 | 154 | 33.48 | 72 | 15.65 | 7 | 1.52 | **460** | **100** |
| **2012** | 249 | 55.21 | 144 | 31.93 | 45 | 9.98 | 13 | 2.88 | **451** | **100** |
| **2013** | 288 | 56.25 | 156 | 30.47 | 61 | 11.91 | 7 | 1.37 | **512** | **100** |
| **2014** | 404 | 57.55 | 208 | 29.63 | 80 | 11.40 | 10 | 1.42 | **702** | **100** |
| **2015** | 401 | 55.77 | 205 | 28.51 | 109 | 15.16 | 4 | 0.56 | **719** | **100** |
| **2016** | 287 | 58.93 | 123 | 25.26 | 76 | 15.61 | 1 | 0.21 | **487** | **100** |
| **2017** | 292 | 60.46 | 135 | 27.95 | 55 | 11.39 | 1 | 0.21 | **483** | **100** |
| **2018** | 538 | 57.97 | 286 | 30.82 | 102 | 10.99 | 2 | 0.22 | **928** | **100** |
| **Total** | **2686** | **56.64** | **1411** | **29.76** | **600** | **12.65** | **45** | **0.95** | **4742** | **100** |

Table 4. Sex-Age distribution in CV AEFI reports

|  | **Age** | | | | | | | | | | | | | |
| --- | --- | --- | --- | --- | --- | --- | --- | --- | --- | --- | --- | --- | --- | --- |
|  | **<1** | | **1-6** | | **7-17** | | **18-64** | | **‎≥65‎** | | **Unknown** | | **Total** | |
| **SEX** | **No.** | **(%)** | **No.** | **(%)** | **No.** | **(%)** | **No.** | **(%)** | **No.** | **(%)** | **No.** | **(%)** | **No.** | **(%)** |
| **Female** | 58 | 1.22 | 174 | 3.67 | 177 | 3.73 | 1295 | 27.31 | 79 | 1.67 | 903 | 19.04 | **2686** | **56.64** |
| **Male** | 87 | 1.83 | 218 | 4.60 | 142 | 2.99 | 466 | 9.83 | 43 | 0.91 | 455 | 9.60 | **1411** | **29.76** |
| **Unknown** | 73 | 1.54 | 161 | 3.40 | 14 | 0.30 | 42 | 0.89 | 3 | 0.06 | 307 | 6.47 | **600** | **12.65** |
| **Not specified** | 10 | 0.21 | 11 | 0.23 | 3 | 0.06 | 7 | 0.15 | - | - | 14 | 0.30 | **45** | **0.95** |
| **Total** | **228** | **4.81** | **564** | **11.89** | **336** | **7.09** | **1810** | **38.17** | **125** | **2.64** | **1679** | **35.41** | **4742** | **100.00** |

Table 5. CV AEFI reports by vaccine combinations, 2011-2018^[[2]](#footnote-3)^

| **Vaccine Combination** | **No.** | **(%)** | **Total (%)** |
| --- | --- | --- | --- |
| VARZOS | 1087 | 22.92% | 22.92% |
| FLU3 | 521 | 10.99% | 33.91% |
| FLUX | 307 | 6.47% | 40.38% |
| PPV | 288 | 6.07% | 46.46% |
| MENB | 256 | 5.40% | 51.86% |
| PNC13 | 159 | 3.35% | 55.21% |
| FLU4 | 142 | 2.99% | 58.20% |
| HPV4 | 135 | 2.85% | 61.05% |
| HEPAB | 130 | 2.74% | 63.79% |
| TDAP | 113 | 2.38% | 66.17% |
| Men-C-C | 99 | 2.09% | 68.26% |
| FLUA3 | 99 | 2.09% | 70.35% |
| Unknown^[[3]](#footnote-4)^ | 95 | 2.00% | 72.35% |
| CHOL | 91 | 1.92% | 74.27% |
| MNQ | 81 | 1.71% | 75.98% |
| VARCEL | 80 | 1.69% | 77.67% |
| RAB | 68 | 1.43% | 79.10% |
| MMR | 60 | 1.27% | 80.37% |
| HPV9 | 59 | 1.24% | 81.61% |
| HEP | 48 | 1.01% | 82.62% |
| TYP | 38 | 0.80% | 83.42% |
| TD | 32 | 0.67% | 84.10% |
| RV1 | 30 | 0.63% | 84.73% |
| DTAPIPVHIB | 26 | 0.55% | 85.28% |
| BCG | 25 | 0.53% | 85.81% |
| HEPA | 23 | 0.49% | 86.29% |
| FLUX+PPV | 23 | 0.49% | 86.78% |
| PNC | 22 | 0.46% | 87.24% |
| DTAPIPV | 21 | 0.44% | 87.68% |
| FLUN3 | 20 | 0.42% | 88.11% |
| FLUN4 | 17 | 0.36% | 88.46% |
| YF | 16 | 0.34% | 88.80% |
| RV5 | 15 | 0.32% | 89.12% |
| DTAPIPVHIB+PNC13 | 13 | 0.27% | 89.39% |
| MMRV | 11 | 0.23% | 89.62% |
| HPV2 | 10 | 0.21% | 89.84% |
| FLUX+VARZOS | 10 | 0.21% | 90.05% |
| 6VAX-F | 10 | 0.21% | 90.26% |
| Other | 462 | 9.74% | 100% |

Table 6. The highly frequent AEs in CV, 2011-2018

| **Adverse Event** | **No.** | **%** | **Total (%)** |
| --- | --- | --- | --- |
| Pyrexia | 452 | 2.51% | 2.51% |
| Headache | 355 | 1.97% | 4.48% |
| Pain in extremity | 339 | 1.88% | 6.37% |
| Fatigue | 320 | 1.78% | 8.15% |
| Herpes zoster | 282 | 1.57% | 9.71% |
| Nausea | 280 | 1.56% | 11.27% |
| Pain | 268 | 1.49% | 12.76% |
| Erythema | 250 | 1.39% | 14.15% |
| Vaccination site erythema | 240 | 1.33% | 15.48% |
| Malaise | 229 | 1.27% | 16.75% |
| Vaccination failure | 220 | 1.22% | 17.98% |
| Injection site pain | 219 | 1.22% | 19.19% |
| Myalgia | 218 | 1.21% | 20.40% |
| Injection site erythema | 216 | 1.20% | 21.60% |
| Vaccination site pain | 205 | 1.14% | 22.74% |
| Dizziness | 199 | 1.11% | 23.85% |
| Pruritus | 190 | 1.06% | 24.91% |
| Vomiting | 185 | 1.03% | 25.93% |
| Diarrhoea | 184 | 1.02% | 26.96% |
| Arthralgia | 180 | 1.00% | 27.96% |
| Drug ineffective | 179 | 0.99% | 28.95% |
| Rash | 172 | 0.96% | 29.91% |
| Vaccination site swelling | 170 | 0.94% | 30.85% |
| Urticaria | 170 | 0.94% | 31.80% |
| Chills | 162 | 0.90% | 32.70% |
| Cough | 156 | 0.87% | 33.56% |
| Dyspnoea | 152 | 0.84% | 34.41% |
| Asthenia | 145 | 0.81% | 35.21% |
| Injection site swelling | 142 | 0.79% | 36.00% |
| Peripheral swelling | 141 | 0.78% | 36.79% |
| Hypoaesthesia | 128 | 0.71% | 37.50% |
| Paraesthesia | 124 | 0.69% | 38.19% |
| Nasopharyngitis | 115 | 0.64% | 38.83% |
| Cellulitis | 112 | 0.62% | 39.45% |
| Vaccination site warmth | 107 | 0.59% | 40.04% |
| Influenza like illness | 102 | 0.57% | 40.61% |
| Other | 10688 | 0.59391 | 100.00% |

Table 7. CV SAE report categories, 2011-2018^[[4]](#footnote-5)^

|  | **2011** | | **2012** | | **2013** | | **2014** | | **2015** | |
| --- | --- | --- | --- | --- | --- | --- | --- | --- | --- | --- |
|  | **No.** | **(%)** | **No.** | **(%)** | **No.** | **(%)** | **No.** | **(%)** | **No.** | **(%)** |
| **Death** | 12 | 5.53 | 9 | 3.67 | 8 | 3.21 | 7 | 1.99 | 9 | 2.56 |
| **Disability** | 18 | 8.29 | 26 | 10.61 | 17 | 6.83 | 36 | 10.26 | 40 | 11.36 |
| **Hospitalization** | 53 | 24.42 | 36 | 14.69 | 79 | 31.73 | 88 | 25.07 | 71 | 20.17 |
| **Life Threatening** | 11 | 5.07 | 11 | 4.49 | 12 | 4.82 | 15 | 4.27 | 10 | 2.84 |
| **Congenital Anomaly** | 2 | 0.92 | 3 | 1.22 | 0 | 0.00 | 0 | 0.00 | 1 | 0.28 |
| **Other Medically Important Condition** | 138 | 63.59 | 185 | 75.51 | 174 | 69.88 | 261 | 74.36 | 276 | 78.41 |
| **Total SAE reports** | **217** | **100** | **245** | **100** | **249** | **100** | **351** | **100** | **352** | **100** |

|  | **2016** | | **2017** | | **2018** | | **Total** | |
| --- | --- | --- | --- | --- | --- | --- | --- | --- |
|  | **No.** | **(%)** | **No.** | **(%)** | **No.** | **(%)** | **No.** | **(%)** |
| **Death** | 8 | 2.42 | 6 | 1.99 | 14 | 2.19 | 73 | 2.72 |
| **Disability** | 31 | 9.37 | 32 | 10.60 | 38 | 5.94 | 238 | 8.86 |
| **Hospitalization** | 59 | 17.82 | 59 | 19.54 | 104 | 16.25 | 549 | 20.43 |
| **Life Threatening** | 11 | 3.32 | 26 | 8.61 | 20 | 3.13 | 116 | 4.32 |
| **Congenital Anomaly** | 1 | 0.30 | 0 | 0.00 | 1 | 0.16 | 8 | 0.30 |
| **Other Medically Important Condition** | 273 | 82.48 | 226 | 74.83 | 548 | 85.63 | 2081 | 77.45 |
| **Total SAE reports** | **331** | **100** | **302** | **100** | **640** | **100** | **2687** | **100** |

Table 8. CV SAE reports by vaccine combinations, 2011-2018^[[5]](#footnote-6)^

| **Vaccine Combination** | **No.** | **(%)** | **Total %** |
| --- | --- | --- | --- |
| VARZOS | 647 | 24.08% | 24.08% |
| FLU3 | 253 | 9.42% | 33.49% |
| FLUX | 238 | 8.86% | 42.35% |
| PPV | 175 | 6.51% | 48.86% |
| HPV4 | 110 | 4.09% | 52.96% |
| HEPAB | 101 | 3.76% | 56.72% |
| Unknown^[[6]](#footnote-7)^ | 83 | 3.09% | 59.81% |
| PNC13 | 82 | 3.05% | 62.86% |
| MENB | 70 | 2.61% | 65.46% |
| TDAP | 62 | 2.31% | 67.77% |
| FLU4 | 55 | 2.05% | 69.82% |
| CHOL | 51 | 1.90% | 71.72% |
| HPV9 | 46 | 1.71% | 73.43% |
| VARCEL | 42 | 1.56% | 74.99% |
| MMR | 38 | 1.41% | 76.40% |
| HEP | 34 | 1.27% | 77.67% |
| RV1 | 27 | 1.00% | 78.68% |
| BCG | 23 | 0.86% | 79.53% |
| TYP | 19 | 0.71% | 80.24% |
| RAB | 19 | 0.71% | 80.95% |
| FLUX-PPV | 17 | 0.63% | 81.58% |
| PNC | 16 | 0.60% | 82.17% |
| Men-C-C | 16 | 0.60% | 82.77% |
| HEPA | 16 | 0.60% | 83.36% |
| FLUA3 | 14 | 0.52% | 83.89% |
| DTAPIPVHIB | 14 | 0.52% | 84.41% |
| RV5 | 13 | 0.48% | 84.89% |
| YF | 12 | 0.45% | 85.34% |
| FLUN3 | 12 | 0.45% | 85.78% |
| DTAPIPV | 12 | 0.45% | 86.23% |
| TD | 11 | 0.41% | 86.64% |
| FLUN4 | 11 | 0.41% | 87.05% |
| 6VAX-F | 10 | 0.37% | 87.42% |
| HPV2 | 9 | 0.33% | 87.76% |
| PNC13+PPV | 8 | 0.30% | 88.05% |
| MNQ | 8 | 0.30% | 88.35% |
| HIBV | 8 | 0.30% | 88.65% |
| FLUX+VARZOS | 7 | 0.26% | 88.91% |
| DTPPHIB | 7 | 0.26% | 89.17% |
| DTAPIPVHIB+PPV | 6 | 0.22% | 89.39% |
| DTAPIPVHIB+PNC13+RV1 | 6 | 0.22% | 89.62% |
| DTAPIPVHIB+PNC13 | 6 | 0.22% | 89.84% |
| PPV+VARZOS | 5 | 0.19% | 90.03% |
| MMRV | 5 | 0.19% | 90.21% |
| HEP+HEPA | 5 | 0.19% | 90.40% |
| Other | 258 | 9.60% | 100.00% |

Table 9. The highly frequent AEs for SAEs in CV, 2011-2018

| **Adverse Event** | **No.** | **%** | **Total (%)** |
| --- | --- | --- | --- |
| Pyrexia | 275 | 2.20% | 2.20% |
| Herpes zoster | 230 | 1.84% | 4.04% |
| Headache | 211 | 1.69% | 5.73% |
| Vaccination failure | 205 | 1.64% | 7.37% |
| Fatigue | 198 | 1.58% | 8.95% |
| Nausea | 169 | 1.35% | 10.30% |
| Pain | 164 | 1.31% | 11.62% |
| Pain in extremity | 161 | 1.29% | 12.90% |
| Malaise | 159 | 1.27% | 14.17% |
| Drug ineffective | 150 | 1.20% | 15.37% |
| Dyspnoea | 135 | 1.08% | 16.45% |
| Dizziness | 132 | 1.06% | 17.51% |
| Cough | 130 | 1.04% | 18.55% |
| Myalgia | 122 | 0.98% | 19.53% |
| Diarrhoea | 118 | 0.94% | 20.47% |
| Arthralgia | 118 | 0.94% | 21.41% |
| Vomiting | 116 | 0.93% | 22.34% |
| Asthenia | 115 | 0.92% | 23.26% |
| Erythema | 109 | 0.87% | 24.13% |
| Pruritus | 104 | 0.83% | 24.97% |
| Urticaria | 102 | 0.82% | 25.78% |
| Injection site pain | 98 | 0.78% | 26.57% |
| Cellulitis | 93 | 0.74% | 27.31% |
| Pneumonia | 92 | 0.74% | 28.05% |
| Nasopharyngitis | 90 | 0.72% | 28.77% |
| Rash | 88 | 0.70% | 29.47% |
| Hypoaesthesia | 82 | 0.66% | 30.13% |
| Chills | 81 | 0.65% | 30.77% |
| Paraesthesia | 76 | 0.61% | 31.38% |
| Injection site erythema | 76 | 0.61% | 31.99% |
| Vaccination site pain | 68 | 0.54% | 32.53% |
| Peripheral swelling | 68 | 0.54% | 33.08% |
| Hypersensitivity | 68 | 0.54% | 33.62% |
| Abdominal pain | 67 | 0.54% | 34.16% |
| Seizure | 66 | 0.53% | 34.69% |
| Oropharyngeal pain | 66 | 0.53% | 35.21% |
| Weight decreased | 63 | 0.50% | 35.72% |
| Condition aggravated | 63 | 0.50% | 36.22% |
| Musculoskeletal pain | 61 | 0.49% | 36.71% |
| Loss of consciousness | 61 | 0.49% | 37.20% |
| Anaphylactic reaction | 61 | 0.49% | 37.68% |
| Influenza | 60 | 0.48% | 38.16% |
| Influenza like illness | 58 | 0.46% | 38.63% |
| Guillain-Barre syndrome | 56 | 0.45% | 39.08% |
| Blood pressure increased | 56 | 0.45% | 39.52% |
| Pneumococcal infection | 55 | 0.44% | 39.96% |
| Facial paralysis | 54 | 0.43% | 40.40% |
| Vaccination site erythema | 50 | 0.40% | 40.80% |
| Syncope | 48 | 0.38% | 41.18% |
| Swelling | 48 | 0.38% | 41.56% |
| Productive cough | 48 | 0.38% | 41.95% |
| Nasal congestion | 48 | 0.38% | 42.33% |
| Chest pain | 48 | 0.38% | 42.72% |
| Gait disturbance | 47 | 0.38% | 43.09% |
| Decreased appetite | 47 | 0.38% | 43.47% |
| Back pain | 47 | 0.38% | 43.84% |
| Hyperhidrosis | 45 | 0.36% | 44.20% |
| Heart rate increased | 45 | 0.36% | 44.56% |
| Rhinorrhoea | 43 | 0.34% | 44.91% |
| Rheumatoid arthritis | 43 | 0.34% | 45.25% |
| Insomnia | 43 | 0.34% | 45.60% |
| Tremor | 42 | 0.34% | 45.93% |
| Injection site swelling | 41 | 0.33% | 46.26% |
| Fall | 41 | 0.33% | 46.59% |
| Asthma | 41 | 0.33% | 46.92% |
| Muscular weakness | 40 | 0.32% | 47.24% |
| Sinusitis | 36 | 0.29% | 47.52% |
| Feeling hot | 36 | 0.29% | 47.81% |
| Chest discomfort | 36 | 0.29% | 48.10% |
| Bronchitis | 36 | 0.29% | 48.39% |
| Pallor | 35 | 0.28% | 48.67% |
| Abdominal pain upper | 35 | 0.28% | 48.95% |
| Vaccination site swelling | 33 | 0.26% | 49.21% |
| Loss of personal independence in daily activities | 33 | 0.26% | 49.48% |
| Haemoglobin decreased | 32 | 0.26% | 49.73% |
| Vision blurred | 31 | 0.25% | 49.98% |
| Urinary tract infection | 31 | 0.25% | 50.23% |
| Hypotension | 31 | 0.25% | 50.48% |
| Muscle spasms | 30 | 0.24% | 50.72% |
| Joint swelling | 30 | 0.24% | 50.96% |
| Visual impairment | 29 | 0.23% | 51.19% |
| Vaccination site warmth | 29 | 0.23% | 51.42% |
| Lymphadenopathy | 29 | 0.23% | 51.65% |
| Weight increased | 28 | 0.22% | 51.88% |
| Musculoskeletal stiffness | 28 | 0.22% | 52.10% |
| Dehydration | 28 | 0.22% | 52.32% |
| Death | 28 | 0.22% | 52.55% |
| Burning sensation | 28 | 0.22% | 52.77% |
| Presyncope | 27 | 0.22% | 52.99% |
| Heart rate decreased | 27 | 0.22% | 53.20% |
| Wheezing | 26 | 0.21% | 53.41% |
| Vaccination site cellulitis | 26 | 0.21% | 53.62% |
| Rash generalised | 26 | 0.21% | 53.83% |
| Neck pain | 26 | 0.21% | 54.04% |
| Blood pressure systolic increased | 26 | 0.21% | 54.24% |
| Swollen tongue | 25 | 0.20% | 54.44% |
| Ophthalmic herpes zoster | 25 | 0.20% | 54.64% |
| Oedema peripheral | 25 | 0.20% | 54.84% |
| Injection site warmth | 25 | 0.20% | 55.04% |
| Arthritis | 25 | 0.20% | 55.24% |
| Other | 5595 | 44.76% | 100.00% |

1. Reports 1 to 8 are the sources for Figure 1. [↑](#footnote-ref-2)
2. For the full name of vaccines please check VAERS DATA USE GUIDE at <https://vaers.hhs.gov/docs/VAERSDataUseGuide_October2017.pdf> [↑](#footnote-ref-3)
3. We were not able to determine vaccine type for 31 vaccine names in CV (total 139 reports). We marked them as Unkown. [↑](#footnote-ref-4)
4. The total sum of SAE categories in each years is more than the total SAE reports in each year. It is because SAE categories are not mutually exclusive and an SAE could have more than one reason of seriousness. [↑](#footnote-ref-5)
5. For the full name of vaccines please check VAERS DATA USE GUIDE at <https://vaers.hhs.gov/docs/VAERSDataUseGuide_October2017.pdf> [↑](#footnote-ref-6)
6. We were not able to determine vaccine type for 31 vaccine names in CV (total 139 reports) so we marked them as Unknown. [↑](#footnote-ref-7)
